# DRD2 Deficiency Underlies Pituitary Adenoma Dependent on *Escherichia coli* Translocation from the Gut

**DOI:** 10.1002/advs.202504247

**Published:** 2026-02-08

**Authors:** Xian‐jun Su, Li Ma, Xi Xiong, Jun‐hua Meng, Qi Wu, Yu Zhang, Shu‐guang Dong, Yue‐fei Wang, Jin‐hu Wu, Qing‐yuan Zeng, Hong‐feng Zhang, Li‐li Li, Liang Meng, Min Peng, Xiao‐dong Huang, Li‐quan Wu, Xiong Wang

**Affiliations:** ^1^ Department of Pharmacy Tongren hospital affiliated to Wuhan University (The Third Hospital of Wuhan) Wuhan China; ^2^ College of Pharmacy Wuhan University of Science and Technology Wuhan China; ^3^ Department of Nuclear Medicine Tongji Hospital Affiliated to Tongji Medical College Huazhong University of Science and Technology Wuhan China; ^4^ Department of Urology surgery Tongren Hospital Affiliated to Wuhan University (The Third Hospital of Wuhan) Wuhan China; ^5^ Department of Pharmacy Pulmonary Hospital of Wuhan Wuhan China; ^6^ Department of Cardiology Tongren Hospital Affiliated to Wuhan University (The Third Hospital of Wuhan) Wuhan China; ^7^ Department of Neurosurgery Tongren Hospital Affiliated to Wuhan University (The Third Hospital of Wuhan) Wuhan China; ^8^ Department of Pathology The central hospital of Wuhan Tongji medical college Huazhong University of Science and Technology Wuhan China; ^9^ Central Laboratory Renmin hospital of Wuhan University Wuhan China; ^10^ Department of Oncology Renmin Hospital of Wuhan University Wuhan China; ^11^ Department of Gastroenterology Tongren hospital affiliated to Wuhan University (The Third Hospital of Wuhan) Wuhan China; ^12^ Department of Neurosurgery Renmin hospital of Wuhan University Wuhan China

**Keywords:** DRD2, *Escherichia coli* translocation, gut, intratumoral microbiome, pituitary adenoma

## Abstract

Pituitary adenoma (PA) are common intracranial tumor types that have harmful effects on human health. However, the pathogenesis of PA remains unclear yet. The intratumoral microbiome has been reported playing an important impact on the occurrence, metastasis, immune monitoring, and drug resistance of various tumors. While normal dopamine receptor D2 (DRD2) expression is enriched in the apical junction of pituitary epithelium and colonic enterocytes, various factors‐induced drd2 loss dampened its expression at both sites. DRD2 deficiencies are characterized by chronic hyperprolactinemia, pituitary lactotroph hyperplasia, and prolactinomas in mice, but the role of intratumoral microbiome in prolactinomas is not known. We employed specific pathogen‐free (SPF) and germ‐free (GF) mice models and patient samples of pituitary adenoma. In the mice pituitary tumor model, we used mice that developed prolactinomas following estradiol treatment or DRD2 deficiencies. Pituitary tumor samples from patients with nonfunctional pituitary adenoma or prolactinomas were obtained after surgical excision. Various molecular, cellular, and sequencing techniques were used to determine the role of intratumoral microbiome in pituitary adenoma. We demonstrate that human patients or murine bearing estradiol‐induction or DRD2 loss are all characterized by the presence of live intratumor bacteria in the pituitary adenoma. Using metagenomic next‐generation sequencing and mass spectrometry techniques, we confirm that the bacterial species of pituitary tumor tissues is *Escherichia coli*. In vitro tracing and immunofluorescence assay results showed that the pathobiont *Escherichia coli* translocates from the gut into the pituitary gland along with DRD2 loss while the blood pituitary barrier were both destroyed in mice. The *Escherichia coli* are phagocytosed by the microglial cells in the pituitary gland, then activate GSDMD protein releasing HMGB1, and promote the tumorigenesis of pituitary adenoma by activating the MAPK pathway. The depletion of bacteria systemically, microglial depletion or HMGB1 inhibitor ethyl pyruvate rescued prolactinomas. Our findings suggest that DRD2 deficiency underlies pituitary adenoma dependent on *Escherichia coli* translocation from the gut and activating microglia GSDMD/ HMGB1/MAPK pathway, and provide a novel preclinical rationale for antimicrobial agents, microglial depletion, or HMGB1 inhibitor ethyl pyruvate for the treatment of pituitary adenoma.

## Graphical abstract

Dopamine receptor D2 (DRD2) deficiency disrupts epithelial barrier integrity in both the gut and pituitary gland, facilitating translocation of gut‐derived *Escherichia coli* into pituitary tissue. Intratumoral E. coli are phagocytosed by microglia, triggering GSDMD‐dependent pyroptosis and HMGB1 release, which subsequently activates MAPK signaling to promote pituitary adenoma progression. Depletion of bacteria, microglia ablation, or inhibition of HMGB1 effectively attenuates tumor development, highlighting a previously unrecognized gutpituitary axis in pituitary adenoma pathogenesis.

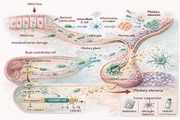

## 1 Introduction

Pituitary adenoma are common intracranial tumor types that have harmful effects on human health [1]. The epidemiological investigation reveals that the morbidity of PA is increasing in recent years, ranging from 10 to 20% in the general population [2].Prolactinomas is the most common type of pituitary tumors, which usually bring serious damage to the body with multiple symptoms, such as galactorrhea, amenorrhea, and infertility [3]. However, the pathogenesis of PA remains unclear yet.

Recently, the intratumoral microbiome has been reported playing important roles in the occurrence, metastasis, immune monitoring, and drug resistance of various tumors. From bone cancer to breast cancer, most tumors bacteria exist in tumors and their adjacent normal tissues, and different kinds of tumors even own unique bacterial communities [4, 5]. There are living resident bacteria in the cytoplasm of breast cancer and they promote the metastasis of breast cancer, and antibiotic gavage therapy can kill intracellular bacteria in tumors and affect tumor metastasis [6, 7]. However, the role of intratumoral microbiome in prolactinomas is not known. DRD2 gene has long been recognized as the therapeutic target of dopamine agonist (DAs) such as bromocriptine (Br) or cabergoline, which reduce pituitary tumor size and diminish redundant prolactin (PRL) levels of prolactinomas patients [8]. However, about 25% of patients with prolactinomas do not respond to DAs therapy [9]. It is usually considered that reduction of pituitary DRD2 expression mediates the resistance to DAs [10, 11]. Previous studies based on data concluded that the DRD2 is highly expressed within the basal ganglia, more specifically the nucleus accumbens and the striatal caudate nucleus [12, 13]. Consequently, the DRD2 expression in other epithelia outside of the pituitary gland and brain has been unknown yet. It is a member of the dopamine receptor family, a group of seven cell transmembrane domain receptors that utilize dopamine as their common ligand [14]. Mice with DRD2 deficiencies are characterized by chronic hyperprolactinemia, pituitary lactotroph hyperplasia and prolactinomas, and have been extensively used as a model of prolactinomas [15, 16]. To date, these abnormalities have not been explained yet.

In this study, we found that DRD2 was expressed both in the pituitary gland epithelium (PGE) and in the gut. The pituitary DRD2 protein expression was gradually decreased significantly during the estradiol induction process in mice. And the formation of cellular junctions between PGE cells in the pituitary gland and between enterocytes in the lower gastrointestinal (GI) tract was damaged by the loss of DRD2. We proved that defective barrier function in the pituitary gland and colon due to DRD2 loss cause translocation of a intestinal pathobiont *Escherichia coli* into the pituitary gland, whereas depletion of bacteria by germ‐free (GF) derivation, or systemic treatment with antibiotics successfully prevented the occurrence and progression of pituitary adenoma. Furthermore, microglial depletion experiments were performed in DRD2(−/−) mice to confirm that the microglia cells of pituitary gland phagocytose bacteria and drive tumorigenesis. Overall, intracellular bacteria activate microglia GSDMD protein, then releasing HMGB1 and promote the tumorigenesis of pituitary adenoma by activating MAPK pathway. Our study first suggest that DRD2 deficiency underlies pituitary adenoma dependent on *Escherichia coli* translocation from the gut.

## 2 Results

### 2.1 Identification of Intratumor Bacteria in Pituitary Gland From Prolactinomas Patients

**FIGURE 1:**
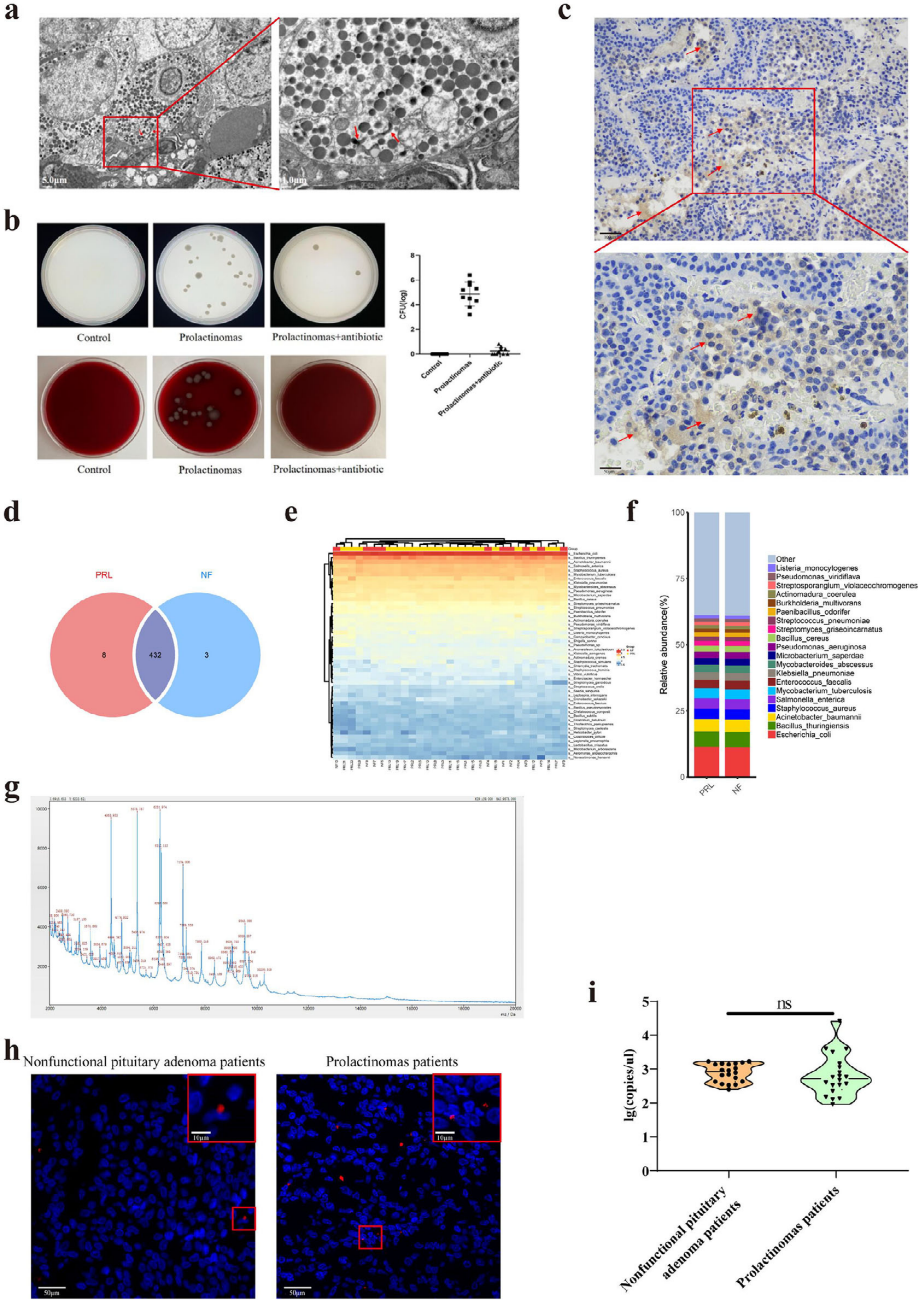
Identification of intratumor bacteria in pituitary gland from prolactinomas patients. (a) Image of bacteria structures within human prolactinomas cell cytosol by TEM. Red arrows refer to bacterial structures. Scale bar, 5.0, 1.0 µm. (b) Bacteria culture and quantification of human prolactinomas tumor tissues with or without antibiotic. n = 10. (c) LPS IHC staining of human prolactinomas tumor section. Scale bar, 100, 50 µm. (d–f) Microbe species number (d), richness histogram (e), and clustering heat map (f) comparison of tumor tissues from ten patients with nonfunctional pituitary adenoma and 21 patients with prolactinomas by microbial metagenomic next‐generation sequencing. (g) Microbial species identification of cultured pituitary tumor‐tissue sample from prolactinomas patients by MS. (h) Detection of *Escherichia coli* in pituitary tumor samples from nonfunctional pituitary adenoma and prolactinomas patients by FISH with *E. coli*‐specific probe. (i) Contents of *Escherichia coli* in pituitary tumor tissues from 20 patients with nonfunctional pituitary adenoma and 20 patients with prolactinomas by FQ‐PCR. NF: Nonfunctional pituitary adenoma, PRL: Prolactinomas. Data were expressed as mean ± S.D. The one‐way ANOVA or Student's t‐test were used to perform statistical comparison by SPSS software. ^*^ *p* < 0.05, ^**^ *p* < 0.01, ^***^ *p* < 0.001, and ^****^ *p* < 0.0001.

We studied the bacteria distribution condition of human prolactinomas primary tumor section by transmission electron microscope (TEM), LPS immunohistochemistry assays (IHC), microbial metagenomic next‐generation sequencing (mNGS) and mass spectrometry (MS) analysis, respectively. The results showed that there are bacteria residing in the cytosol of pituitary prolactin‐producing tumor cells by transmission electron microscope (TEM) assay (Figure 1a), and they were Gram‐negative bacteria in the tumor tissues by LPS IHC (Figure 1c). Furthermore, the dissociated human prolactinomas tumor sample were used to culture bacterial in Mueller–Hinton agar or blood agar plate with or without antibiotic, respectively, showing that there were massive of live bacteria in the tumor cells. The same experimental phenomena was observed in tumor tissues from ten patients with prolactinomas (positive result rate is 100%) (Figure 1b). To identify the bacteria species, the tumor‐tissue specimens from ten patients with nonfunctional pituitary adenoma and 21 patients with prolactinomas were measured by mNGS. The results revealed that the highest relative abundance of bacteria was *Escherichia coli* (Figure 1d–f). Furthermore, the cultured pituitary tumor‐tissue sample from nonfunctional pituitary adenoma and prolactinomas patients were examined by MS for the identification of microbial species, and confirmed they were only *Escherichia coli* (Figure 1g). And *Escherichia coli* in pituitary tumor samples from nonfunctional pituitary adenoma and prolactinomas patients were detected by FISH with *E. coli*‐specific probe, showing that *Escherichia coli* both existing in tumor samples of nonfunctional pituitary adenoma and prolactinomas patients (Figure 1h). The contents of *Escherichia coli* in pituitary tumor samples from 20 patients with nonfunctional pituitary adenoma and 20 prolactinomas patients were also determined by fluorescence quantitative PCR (Figure 1i).

### 2.2 Intratumor Bacteria of Pituitary Gland Along With DRD2 Loss in Prolactinomas Mice

**FIGURE 2:**
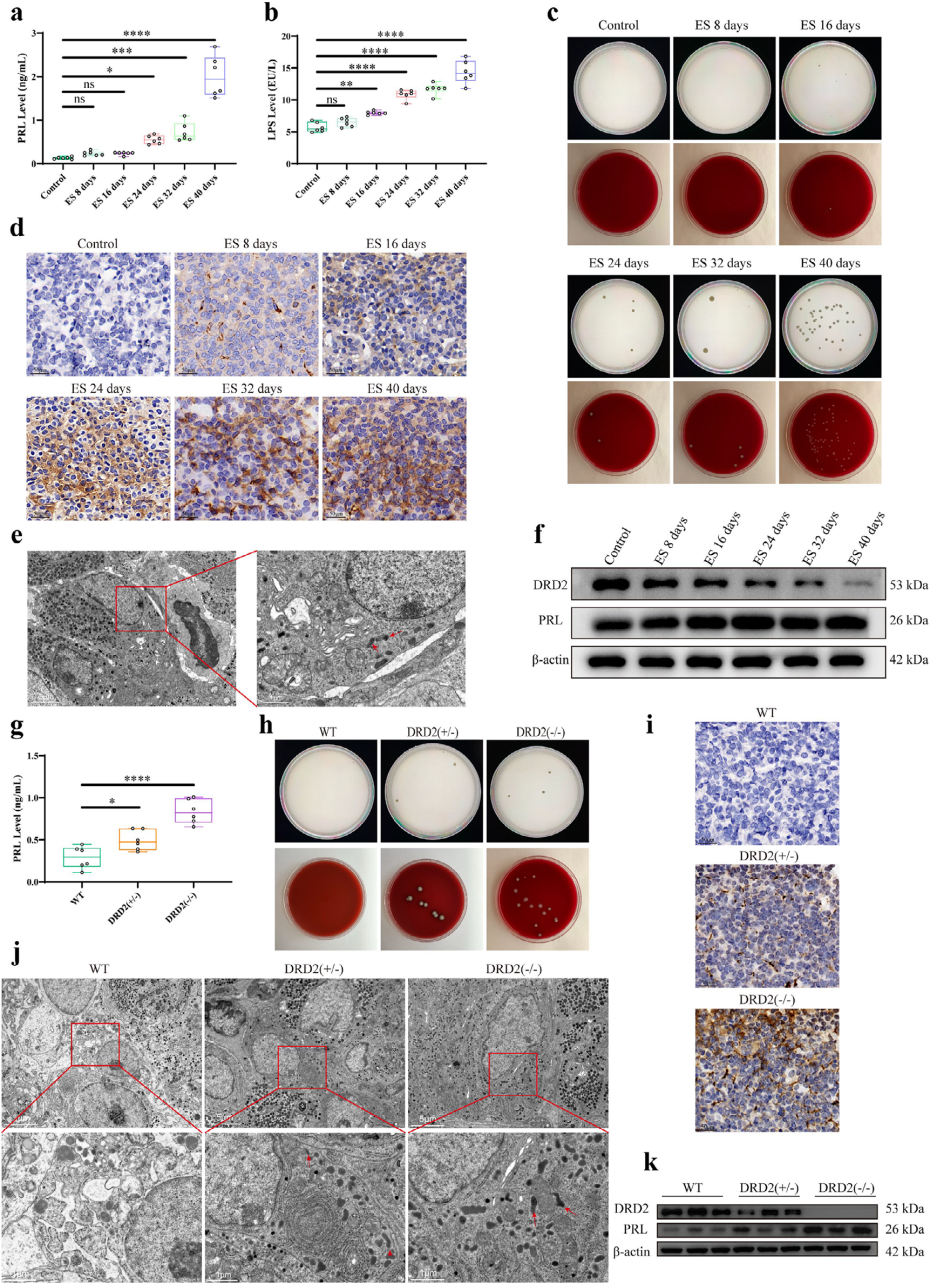
Intratumor bacteria of pituitary gland along with DRD2 loss in prolactinomas mice. (a, b) Serum PRL, LPS levels of control and prolactinomas mice induced by estradiol induction at 8, 16, 24, 32, and 40 days, respectively, by ELISA. n = 6. (c) Bacteria culture of pituitary tissue from control and prolactinomas mice induced by estradiol induction at 8, 16, 24, 32, and 40 days, respectively. n = 3. (d) LPS IHC staining of pituitary tissue from control and prolactinomas mice induced by estradiol induction at 8, 16, 24, 32, and 40 days, respectively. Blue, DAPI. Scale bar, 50 µm. (e) Image of bacteria structures within prolactinomas cell cytosol from mice induced by estradiol induction by TEM. Red arrows refer to bacterial structures. Scale bar, 5.0 µm, 1.0 µm. (f) DRD2 and PRL protein expression of pituitary tissue from control and prolactinomas mice induced by estradiol induction at 8, 16, 24, 32, and 40 days, respectively, by Western blot. n = 3. (g) Serum PRL level of wild type or DRD2 deficiency (± or −/−) mice by ELISA. n = 6. (h) Bacteria culture of pituitary tissue from wild type or DRD2 knockout(± or −/−) mice. n = 3. (i) LPS IHC staining of pituitary tissue from wild type or DRD2 knockout(± or −/−) mice. Blue, DAPI. Scale bar, 50 µm. (j) Image of bacteria structures within prolactinomas cell cytosol from wild type or DRD2 knockout (± or −/−) mice by TEM. Red arrows refer to bacterial structures. Scale bar, 5.0, 1.0 µm. (k) DRD2 and PRL protein expression of pituitary tissue from wild type or DRD2 knockout(± or −/−) mice by Western blot. n = 3. ^*^ *p* < 0.05, ^**^ *p* < 0.01, ^***^ *p* < 0.001, and ^****^ *p* < 0.0001. Data were expressed as mean ± S.D. The one‐way ANOVA or Student's t‐test were used to perform statistical comparison by SPSS software. ^*^ *p* < 0.05, ^**^ *p* < 0.01, ^***^ *p* < 0.001, and ^****^ *p* < 0.0001.

Estradiol induction or DRD2 knockout mice have been widely used as prolactinomas animal model. Although DRD2 has been recognized associated with prolactinomas, how it contribute to this phenotype has remained unknown. We therefore investigated the DRD2 expression of the pituitary gland in ES‐induced and DRD2 knockout mice. We found that pituitary DRD2 protein expression were gradually decreased at 8, 16, 24, 32, and 40 days, and serum and pituitary PRL expression were both increased significantly during the 40 days estradiol induction process (Figure 2a,f). Furthermore, we studied the bacteria distribution condition of mice prolactinomas primary tumor section by LPS IHC analysis, respectively. The results revealed that there were both bacteria in the tumor tissues whenever estradiol induction or DRD2 deficiency (DRD2 ± or DRD2 −/−) mice (Figure 2d,i). Moreover, these bacteria predominantly resided in the cytosol of pituitary prolactin‐producing tumor cells by transmission electron microscope (TEM) assay (Figure 2e,j). Then, the dissociated tumor sample of prolactinomas mice were used to culture bacterial in Mueller–Hinton agar or blood agar plate, respectively, showing that there were massive of bacteria in the tumor cells (Figure 2c,h). The same experimental phenomena were observed in tumor tissues from six prolactinomas mice induced by estradiol intervention or DRD2 knockout (positive result rate is 100%).

### 2.3 Reversal of Pituitary Adenoma in Prolactinomas Mice Through Manipulation of Systemic Bacteria by Germ‐Free Derivation

**FIGURE 3:**
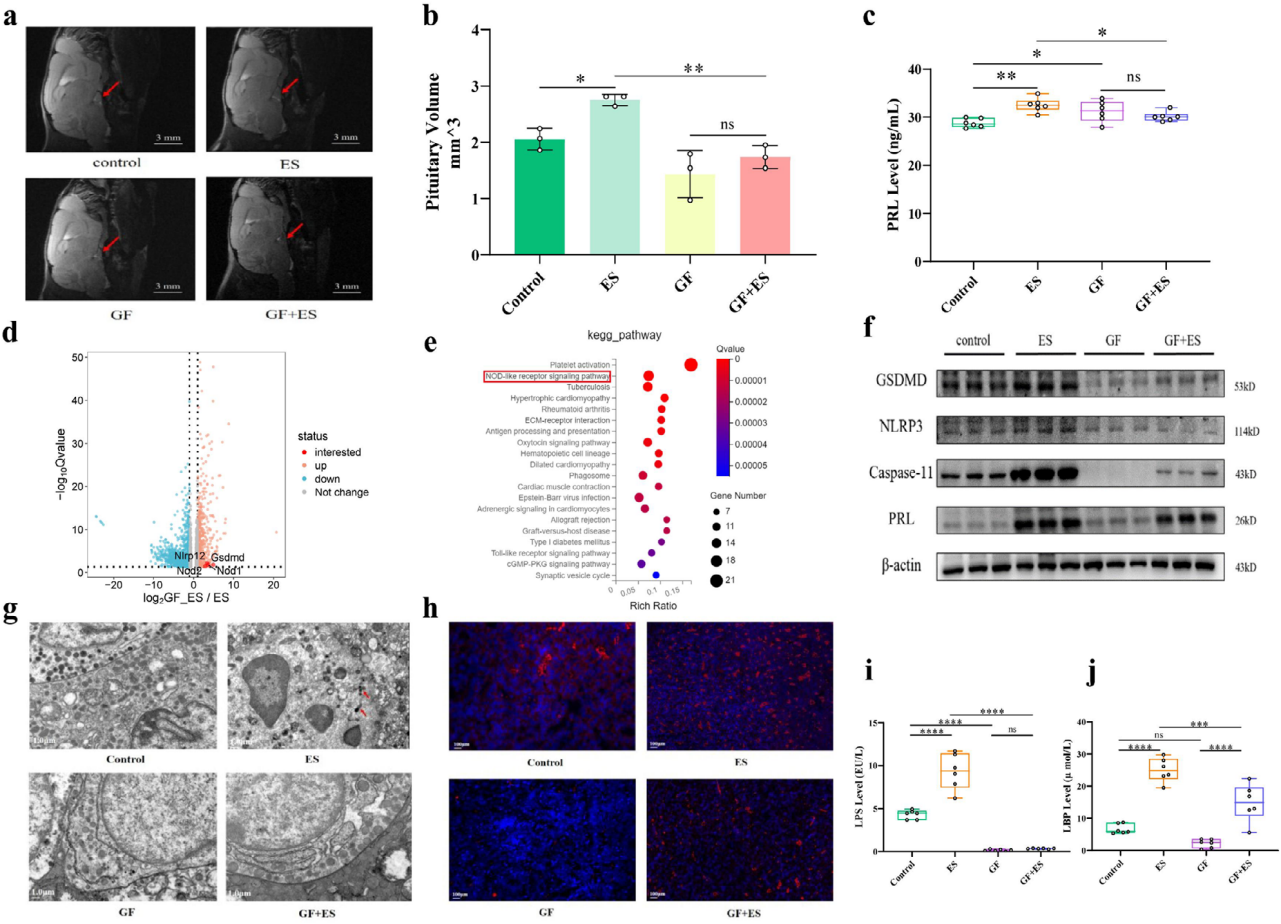
Reversal of pituitary tumor in prolactinomas mice through manipulation of systemic bacteria by germ‐free condition. (a, b) Pituitary tumor volumes of mice were determined by NMR. n = 3. (c) Serum PRL level of mice were determined by ELISA. n = 6. (d, e) Differential top genes and signaling pathway of pituitary tissue between GF mice and SPF mice induced by ES by RNA sequencing. n = 6. (f) PRL and GSDMD‐mediated pyroptosis proteins of pituitary tissue in mice by Western blot. n = 3. (g) Bacteria structures within cytosol in pituitary tissues of mice by TEM. Red arrows refer to bacterial structures. Scale bar, 1.0 µm. (h) GSDMD protein expression of pituitary tissue in mice were determined by immunofluore‐scence assay. Scale bar, 100 µm. (i, j) Serum LPS and LBP levels of mice were determined by ELISA. n = 6. ^*^ *p* < 0.05, ^**^ *p* < 0.01, ^***^ *p* < 0.001, and ^****^ *p* < 0.0001. ES: estradiol, GF: germ‐free, SPF: specific pathogen‐free. Data were expressed as mean ± S.D. The one‐way ANOVA or Student's t‐test were used to perform statistical comparison by SPSS software. ^*^ *p* < 0.05, ^**^ *p* < 0.01, ^***^ *p* < 0.001, and ^****^ *p* < 0.0001.

To confirm the role of bacteria in the pituitary adenoma, germ‐free (GF) mice were used to evaluate after continuous estradiol induction. Nuclear magnetic resonance (NMR) testing showed that the pituitary tumor volume of GF mice was diminished significantly than that of specific pathogen‐free mice (SPF) mice after ES induction (Figure 3a,b). Compared with SPF mice, the serum and pituitary PRL protein expression were much lower in GF mice induced by ES (*p *< 0.05, Figure 3c,f), as same as serum LPS (*p *< 0.0001) and LBP (*p *< 0.05) levels (Figure 3i,j). And bacteria were only found in the cytosol of pituitary gland tissue in SPF mice induced by ES, while no bacteria in control mice, GF mice and GF mice induced by ES (Figure 3g). To illustrate the mechanism of intratumor bacteria promoting pituitary tumor growth and PRL expression, the differential signaling pathway of pituitary tissues between GF mice and SPF mice induced by ES were analysed by RNA sequencing. KEGG pathway analysis showed that NOD‐like receptor signaling pathway was enriched significantly (Figure 3d,e). Furthermore, pituitary NLRP3, Caspase‐11, and GSDMD protein expressions of GF mice were diminished significantly than those of SPF mice induced by ES (*p *< 0.05, Figure 3f). And GSDMD protein expression of pituitary tissue in mice was also determined and verified by immunofluorescence assay (Figure 3h). These data suggest that intratumor bacteria play oncogenesis role in pituitary adenoma by activating GSDMD‐mediated pyroptosis pathway.

### 2.4 Inflammatory Cytokines and Pituitary or Colon Bacteria Contents in Prolactinomas Mice

**FIGURE 4:**
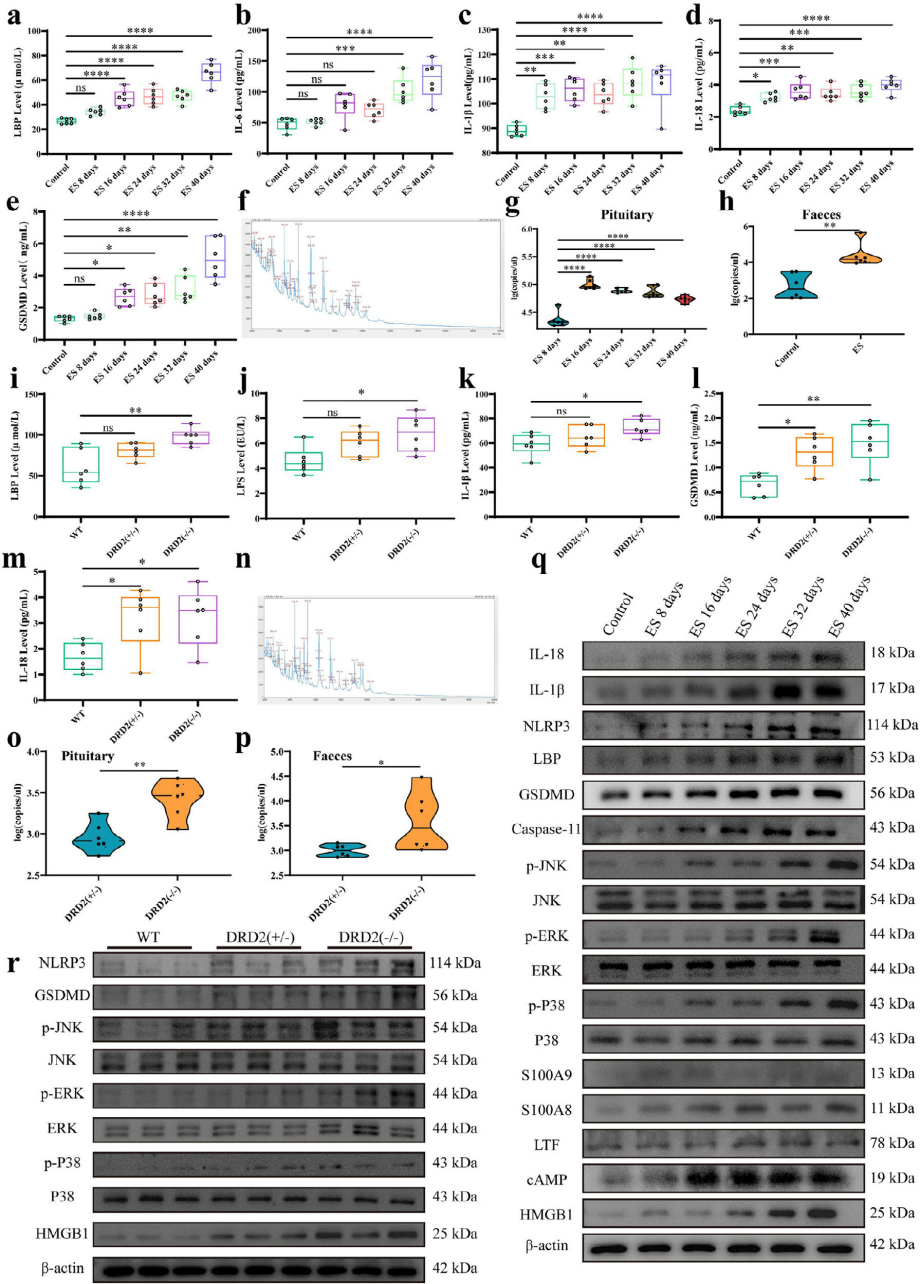
Inflammatory cytokines and pituitary or colon bacteria contents in prolactinomas mice. (a–e) Serum LBP, IL‐6, IL‐1β, IL‐18, and GSDMD levers of prolactinomas mice induced by ES induction at 8, 16, 24, 32, and 40 days, respectively, by ELISA. n = 6. (f) Microbial species identification of cultured pituitary tumor‐tissue sample from prolactinomas mice induced by ES at 8, 16, 24, 32, and 40 days, respectively, by MS. (g) Pituitary contents of *Escherichia coli* in prolactinomas mice induced by ES at 8, 16, 24, 32, and 40 days, respectively by FQ‐PCR. n = 6. (h) Faece contents of *Escherichia coli* in prolactinomas mice induced by ES at 40 day by FQ‐PCR. n = 3. (i–m) Serum LBP, LPS, IL‐1β, IL‐18 and GSDMD levers in DRD2(−/−) mice by ELISA. n = 6. (n) Microbial species identification of cultured pituitary tumor‐tissue sample from DRD2(−/−) mice by MS. (0) Pituitary contents of *Escherichia coli* in DRD2(−/−) mice by FQ‐PCR. n = 6. (p) Faece contents of *Escherichia coli* in DRD2(−/−) mice by FQ‐PCR. n = 6. (q) Pituitary inflammatory cytokines and MAPK signaling proteins of prolactinomas mice induced by ES at 8, 16, 24, 32, and 40 days, respectively, by Western blot. n = 3. (r) Pituitary inflammatory cytokines and MAPK signaling proteins of DRD2(±) and DRD2(−/−) mice by Western blot. n = 3. Data were expressed as mean ± S.D. The one‐way ANOVA or Student's t‐test were used to perform statistical comparison by SPSS software. ^*^ *p* < 0.05, ^**^ *p* < 0.01, ^***^ *p* < 0.001, and ^****^ *p* < 0.0001.

Serum and pituitary inflammatory cytokines levels of mice induced by ES induction at 8, 16, 24, 32, and 40 days were determined, respectively. Compared with control mice, serum LBP (*p *< 0.0001), IL‐6 (*p *< 0.0001), IL‐1β (*p *< 0.0001), IL‐18 (*p *< 0.0001), and GSDMD (*p *< 0.0001) levers of mice induced by ES induction increased gradually at 8, 16, 24, 32, and 40 days (Figure 4a–e). Similarly, pituitary NLRP3 (*p *< 0.05), Caspase‐11 (*p *< 0.05), LBP (*p *< 0.05), IL‐1β (*p *< 0.05), IL‐18(*p *<0.05), and GSDMD (*p *<0.05) protein expressions of mice induced by ES induction were also increased gradually at 8, 16, 24, 32, and 40 days than those of control mice (Figure 4q). The serum and pituitary inflammatory cytokines protein of mice with DRD2 deficiency were also determined. Compared with wild type (WT) mice, serum LBP (*p *<0.01), LPS (*p *<0.05), IL‐1β (*p *<0.05), IL‐18 (*p *<0.05), and GSDMD (*p *<0.01) levers of mice were increased in DRD2(−/−) mice (Figure 4i–m). Similarly, pituitary NLRP3(*P *<0.05) and GSDMD (*p *<0.05) protein expressions were all elevated significantly in DRD2(−/−) mice (Figure 4r). Furthermore, pituitary bacteria *Escherichia coli* were both identified in ES‐induced or DRD2(−/−) mice by MS (Figure 4f–n). And the pituitary contents of *Escherichia coli* in mice induced by ES induction at 8, 16, 24, 32, and 40 days or DRD2(−/−) mice were all determined by FQ‐PCR separately (Figure 4g,o). And faece contents of *Escherichia coli* in mice induced by ES induction at 40 day or DRD2(−/−) mice were also determined (Figure 4h,p). HMGB1 released from pyroptotic cells induces tumor cell proliferation and PCNA expression, and participates in the tumorigenesis of various types of tumors through the ERK1/2 pathway [21, 22, 23]. Therefore, GSDMD/HMGB1/MAPK signaling pathway were also determined in the pituitary tissue of mice induced by ES induction at 8, 16, 24, 32, and 40 days or DRD2(−/−) mice, respectively. The results showed that pituitary HMGB1, p‐JNK/JNK, p‐ERK/ERK, and p‐P38/P38 in mice induced by ES induction at 8, 16 (*p *< 0.05), 24 (*p *< 0.05), 32 (*p *< 0.05) and 40 (*p *< 0.01) days or in DRD2(−/−) (*p *< 0.01) mice were increased significantly than those of control mice (Figure 4r). These results suggest that GSDMD/HMGB1/MAPK signaling pathway participates in the tumorigenesis of prolactinomas.

### 2.5 Reversal of Pituitary Tumor in Prolactinomas Mice Through GSDMD Deficiency

**FIGURE 5:**
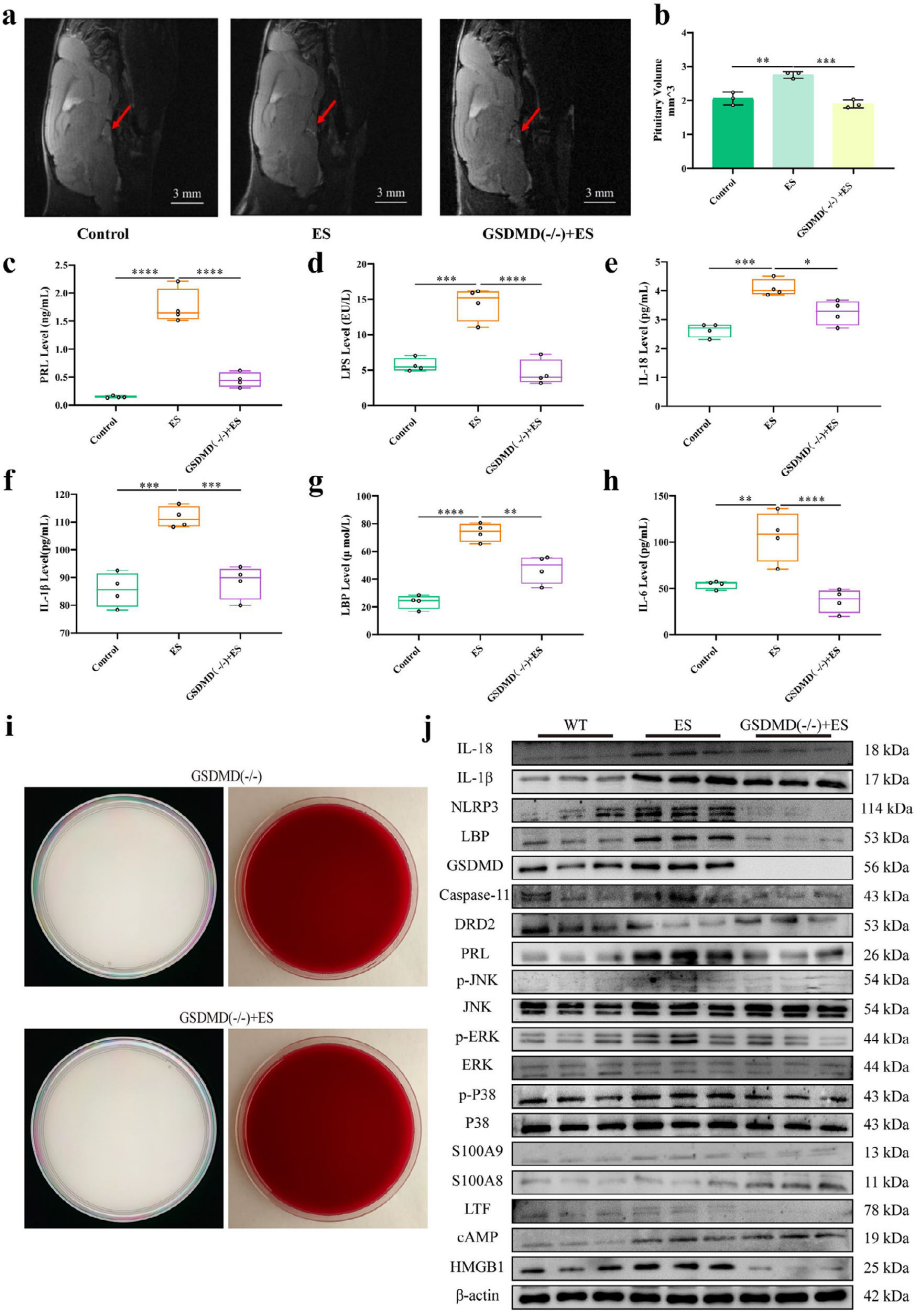
Reversal of pituitary tumor in prolactinomas mice through GSDMD deficiency. (a, b) Pituitary tumor growth volume was determined in mice by NMR. (c) Serum PRL level was determined in mice by ELISA. n = 6. (d–h) Serum inflammatory cytokines LPS, LBP, IL‐1β, IL‐18, and IL‐6 levels were determined in mice by ELISA. n = 6. (i) Bacteria culture of dissociated pituitary tissue in GSDMD(−/−) mice with or without ES induction. (j) GSDMD/HMGB1/MAPK pathway protein expression of pituitary tissue in mice by Western blot. n = 3. Data were expressed as mean ± S.D. The one‐way ANOVA or Student's t‐test were used to perform statistical comparison by SPSS software. ^*^ *p* < 0.05, ^**^ *p* < 0.01, ^***^ *p* < 0.001, and ^****^ *p* < 0.0001.

To further confirm the role and mechanism of GSDMD‐mediated inflammatory signaling pathway in prolactinomas, GSDMD knockout mice were treated with ES induction. Compared with ES‐induced mice, pituitary tumor volume of GSDMD(−/−) mice induced by ES for 40 days was decreased significantly (Figure 5a,b, *p *< 0.001). Serum PRL level of GSDMD(−/−) mice was reduced significantly than that of mice induced by ES (Figure 5c, *p *< 0.0001), as same as inflammatory factors LPS (Figure 5d, *p *<0.0001), IL‐18 (Figure 5e, *p *<0.05), IL‐1β (Figure 5f, *p *<0.001), LBP (Figure 5g, *p *<0.01), IL‐6 (Figure 5h, *p*<0.0001) levels. Mechanismlly, there were no bacteria of dissociated pituitary tissues in GSDMD(−/−) mice with or without ES induction (Figure 5i). And pituitary GSDMD/HMGB1/MAPK pathway protein expressions of GSDMD (−/−) mice were all decreased significantly than those of mice after induced by ES (Figure 5j, *p *<0.01). These data suggested that pituitary bacteria promote the formation of prolactinomas by activating GSDMD/HMGB1/MAPK signaling pathway.

### 2.6 Reversal of Pituitary Adenoma in Prolactinomas Mice Through Manipulation of Systemic Bacteria by Antibiotics Treatment or HMGB1 Inhibitor

**FIGURE 6:**
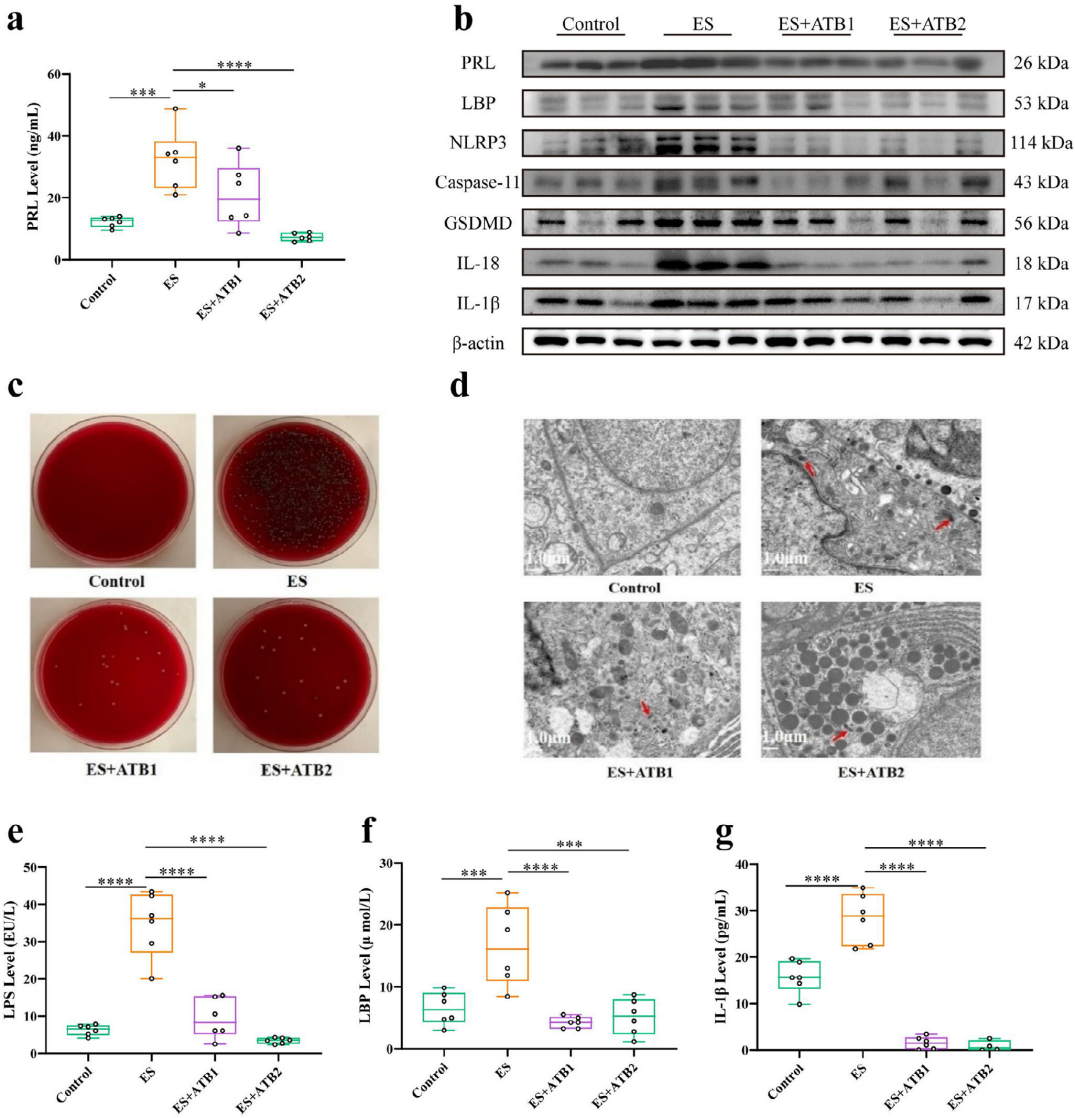
Reversal of pituitary adenoma in prolactinomas mice through manipulation of systemic bacteria by antibiotics treatment. (a) Serum PRL level was determined in prolactinomas mice treated with ATB1 or ATB2 by ELISA. n = 6. (b) PRL and GSDMD‐mediated pyroptosis protein of pituitary tissue were detected in prolactinomas mice treated with ATB1 or ATB2 by Western blot. n = 6. (c) Bacteria culture of prolactinomas tumor cells in mice treated with ATB1 or ATB2. (d) Bacteria structures within cytosol in pituitary tissues of mice treated with ATB1 or ATB2 by TEM. Red arrows refer to bacterial structures. Scale bar, 1.0 µm. (e–g) Serum LPS, LBP, or IL‐1β levels were determined in prolactinomas mice treated with ATB1 or ATB2 by ELISA. n = 6. ^*^ *p* < 0.05, ^**^ *p* < 0.01, ^***^ *p* < 0.001, and ^****^ *p* < 0.0001. ATB1: Combined treatment of vancomycin, Imipenem/Cilastatin Sodium, neomycin, and amphotericin; ATB2: Combined treatment of doxycycline, clarithromycin, and azithromycin. Data were expressed as mean ± S.D. The one‐way ANOVA or Student's t‐test were used to perform statistical comparison by SPSS software. ^*^ *p* < 0.05, ^**^ *p* < 0.01, ^***^ *p* < 0.001, and ^****^ *p* < 0.0001.

We studied the effect of antibiotics treatment on bacteria growth and PRL expression in prolactinomas mice induced by ES. Two kinds of antibiotic combination (ATB1 or ATB2) were administered to ES‐induced mice by oral gavage for 30 days individually. Compared with the ES mice, serum PRL level (Figure 6a, *p *< 0.05) and pituitary PRL protein (Figure 6b, *p *< 0.05) were both significantly decreased in ES‐induced mice treated by ATXB1 or ATXB2, as same as pituitary NLRP3, Caspase‐11, GSDMD, IL‐1β, and IL‐18 proteins (Figure 6b, *p *< 0.05). Pituitary bacteria of mice induced by ES after treated with ATB1 or ATB2 decreased significantly by bacteria culture (Figure 6c). And TEM assay results showed that bacteria of pituitary gland tissue in antibiotics‐treated mice were less than that in ES‐induced mice significantly (Figure 6d). Similarly, serum LPS (*p *< 0.0001), LBP (*p *< 0.001), and IL‐1β (*p *< 0.0001) levels of mice induced by ES were all also reversed by ATXB1 or ATXB2 treatment remarkably (Figure 6e–g). More, another experiment using narrow‐spectrum antibiotics aztreonam was performed to study the role of *E. coli* in tumorigenesis. In clinical practice, aztreonam is mainly used to kill Gram's negative bacteria such as *E. coli* and so on. In our experiment, aztreonam gavage decreased pituitary PRL, NLRP3, HMGB1, IL‐1β, and IL‐18 levels significantly in DRD2(−/−) prolactinomas mice once a day for 10 days. These results showed that *E. coli* is the primary driver of tumorigenesis (Figure S1a). In addition, the HMGB1 inhibitor ethyl pyruvate solution was injected intraperitoneally into DRD2(−/−) prolactinomas mice once a day for 10 days. The results showed that pituitary PRL, NLRP3, GSDMD, and HMGB1 protein expressions were decreased by ethyl pyruvate treatment in DRD2(−/−) prolactinomas mice, suggesting that *Escherichia coli* activates the GSDMD/HMGB1/MAPK signaling pathway in pituitary tumors (Figure S1b).

### 2.7 Oncogenesis Role of Intracellular *Escherichia coli* on Pituitary Tumor In Vitro

**FIGURE 7:**
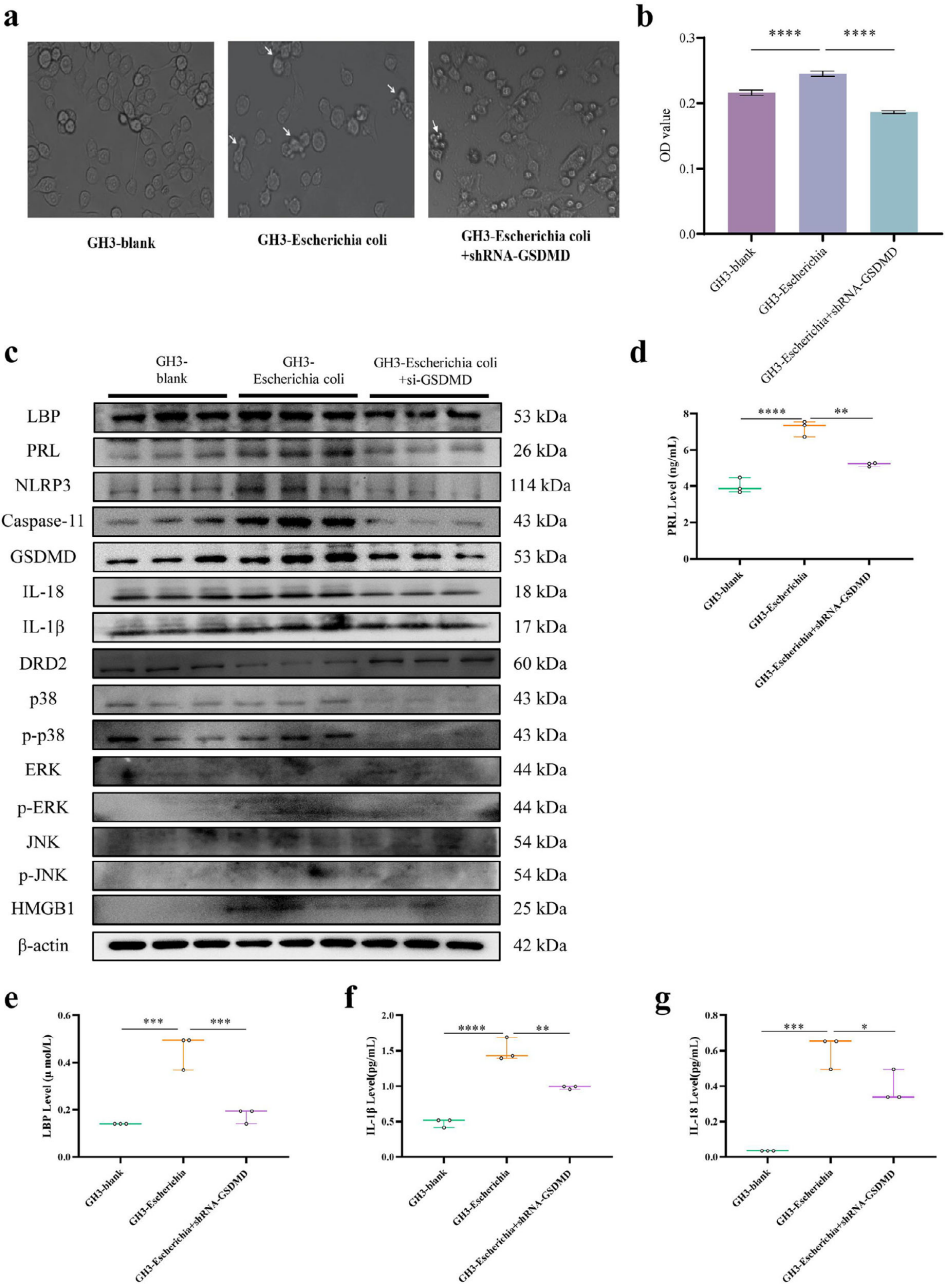
Oncogenesis role of intracellular *Escherichia coli* on pituitary tumor in vitro. (a) Cell morphology of GH3 cells after *Escherichia coli* electroporation with or without GSDMD knockdown at 3 h by electronic microscopes (magnification 10x). (b) Cell proliferation of GH3 cells after *Escherichia coli* electroporation with or without GSDMD knockdown at 3 h by CCK8. (c) PRL, DRD2, and GSDMD‐mediated HMGB1/MAPK pathway protein expression of GH3 cell lysates by Western blot. n = 3. (d) Supernatant PRL level of GH3 cells after *Escherichia coli* electroporation with or without GSDMD knockdown at 3 h by ELISA. n = 3. (e–g) LBP, IL‐1β, or IL‐18 protein levels of GH3 cell culture supernatants at 3 h by ELISA. n = 3. Data were expressed as mean ± S.D. The one‐way ANOVA or Student's t‐test were used to perform statistical comparison by SPSS software. ^*^ *p* < 0.05, ^**^ *p* < 0.01, ^***^ *p* < 0.001, and ^****^ *p* < 0.0001.

Further study was performed to study the regulatory mechanism of *Escherichia coli* in the cytosol of pituitary gland cells in vivo. It was found that the pituitary tumor cell proliferated significantly after *Escherichia coli* electroporated into GH3 cells at 3 h (Figure 7a,b, *p *< 0.05). The PRL, LBP, NLRP3, Caspase‐11, GSDMD, IL‐1β, IL‐18, HMGB1, p‐ERK/ERK, and p‐P38/P38 protein expression of cell lysates were all increased while DRD2 protein expression decreased significantly after *Escherichia coli* electroporated into GH3 cells (*p *< 0.05), which were reversed by GSDMD knockdown (Figure 7c, *p *< 0.05). Meanwhile, the PRL (*p *< 0.0001), LBP (*p *< 0.001), IL‐1β (*p *< 0.0001), and IL‐18 (*p *< 0.001) levels were elevated significantly while decreased in GSDMD‐knockdown GH3 cell culture supernatants after *Escherichia coli* electroporation (Figure 7d–g, *p *< 0.05). These results illustrate that *Escherichia coli* promotes the proliferation of pituitary tumor cell and PRL expression significantly by activating GSDMD‐mediated HMGB1/MAPK signaling pathway. Similarly, co‐culture experiments with pituitary cells and *E. coli* was conducted. The results showed that *E. coli* promoted pituitary cells proliferation significantly by CCK8 experiments. And more, it is found that MAPK protein (p38, ERK, and JNK) expressions all increased markedly in GH3 cells co‐cultured with *E. coli*, suggesting that *E. coli* directly induces tumorigenesis (Figure S2).

### 2.8 Damage of Blood Pituitary Barrier in Prolactinomas Mice

**FIGURE 8:**
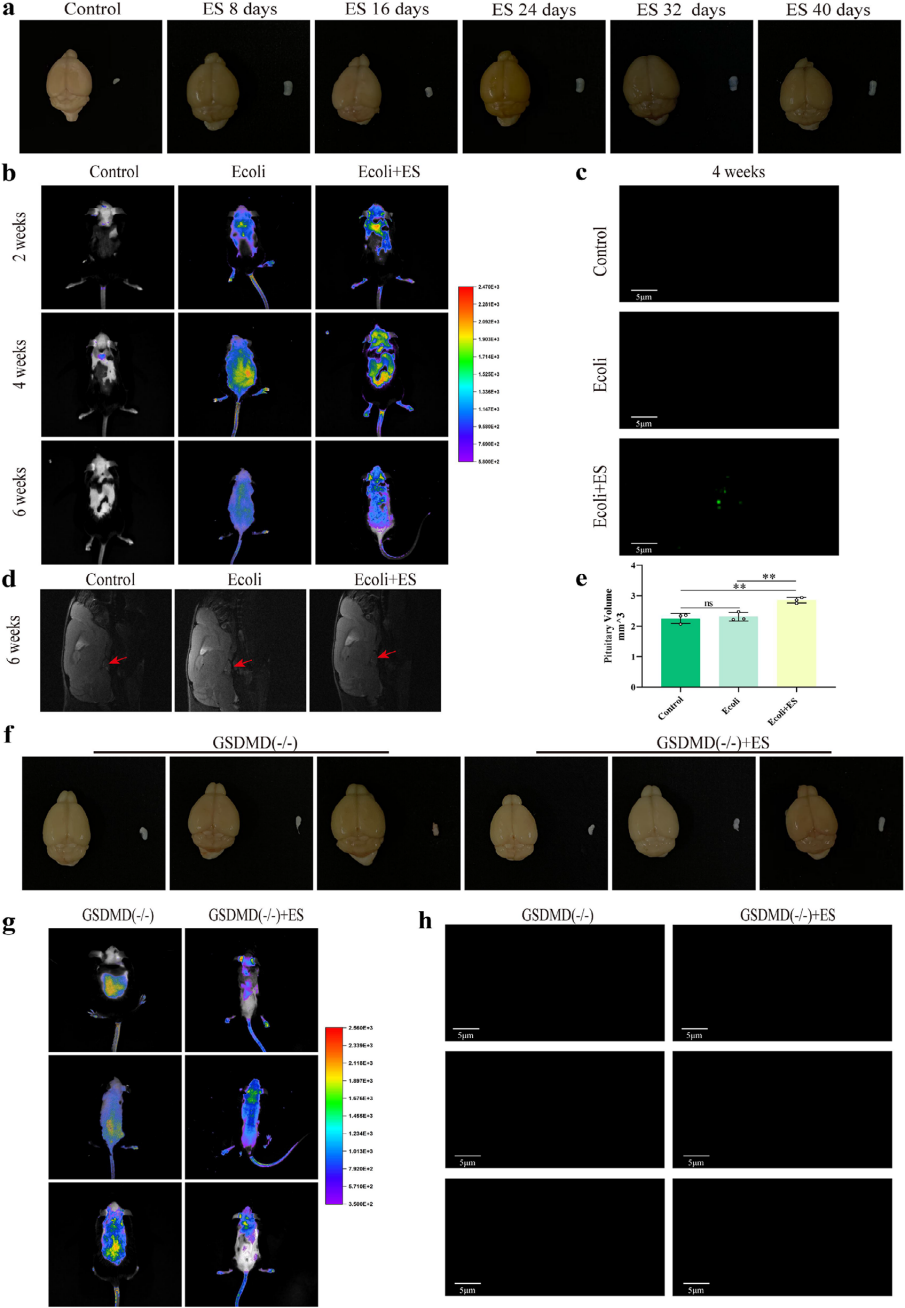
Damage of blood pituitary barrier in prolactinomas mice. (a) BPB defect change of pituitary gland in mice after ES induction at 8, 16, 24, 32, and 40 days by observing evans blue color. Left: brain tissue, right: pituitary gland. (b) In vitro tracing of *Escherichia coli*. labeled with fluorescent in mice with or without ES induction. n = 6. (c) Pituitary *Escherichia coli*. determination by immunofluorescence assay in mice with or without ES induction. n = 6. (d, e) NMR detection of pituitary tumor in mice after *Escherichia coli*. injection with or without ES. n = 3. (f) BPB defect change of pituitary gland in GSDMD(−/−) mice with or without ES at 40 days by observing evans blue color. Left: brain tissue, right: pituitary gland (g) In vitro tracing of *Escherichia coli*. labeled with fluorescent in GSDMD(−/−) mice with or without ES for 40 days. n = 6. (h) Pituitary *Escherichia coli*. determination by immunofluorescence assay in GSDMD(−/−) mice with or without ES for 40 days. n = 6. Data were expressed as mean ± S.D. The one‐way ANOVA or Student's t‐test were used to perform statistical comparison by SPSS software. ^*^ *p* < 0.05, ^**^ *p* < 0.01, ^***^ *p* < 0.001, and ^****^ *p* < 0.0001.

To explore whether the blood pituitary barrier (BPB) was damaged in prolactinomas mice, Evans blue was injected into the tail vein of mice to monitor the BPB leakage [24]. We found that bacteria challenge caused massive extravasation of Evans blue dye in whole pituitary glands of wild‐type mice treated by ES induction for 8, 16, 24, 32, and 40 days, respectively. The result revealed that the color of pituitary gland became deeper gradually along with the time increased, suggesting the blood pituitary barrier of mice was destroyed seriously in the process of ES injection (Figure 8a). To further study the effect of bacteria on blood brain barrier, *Escherichia coli*. labeled with fluorescent was injected into the abdominal cavity of mice with or without ES, respectively. In vivo tracing and immunofluorescence assay results showed that *Escherichia coli*. entered the pituitary tissue of mice after 4 weeks of ES injection (Figure 8b,c). Similarly, the blood pituitary barrier were both destroyed, and *Escherichia coli*. from gut entered the pituitary tissue in whenever heterozygous (DRD2 ±) or knockout (DRD2 −/−) mice (Figure S3a–c). NMR detection showed that the pituitary tumor volume of mice treated with ES and *Escherichia coli*. was increased significantly than those of control or mice treated with *Escherichia coli*.(Figure 8d,e). GSDMD integrate various inflammatory stimuli in brain endothelial cell (bECs), resulting in blood–brain barrier (BBB) leakage and brain inflammation [24]. Therefore, GSDMD (−/−) mice was injected with ES for 40 days to evaluate the role of GSDMD in BPB. The results revealed that the color of pituitary glands were not changed yet (Figure 8f), and *Escherichia coli*. did not enter the pituitary tissue after ES injection by in vitro tracing and immunofluorescence assay (Figure 8g,h). These results illustrate that *Escherichia coli*. from gut entered the pituitary tissue in mice induced by ES through damaging BPB, which was reversed by GSDMD deficiency. To confirm the *Escherichia coli*. distribution in colon, mice were treated with intraperitoneal injection of ES. The results showed that fluorescent *E. coli* was inside the colon (including apical side and basal side) of mice (Figure S4a). And to further demonstrate the origin of *E. coli*, *E. coli* labeled with fluorescent was injected into the abdominal cavity of mice with or without ES, respectively. The results showed that fluorescent *E. coli* was also inside the colon (including apical side and basal side) of mice and was much more than that of mice treated only with ES (Figure S4b), suggesting that *E. coli* could penetrate both the apical and basal sides of intestinal epithelial cells. These data demonstrated that *E. coli* penetrated into the colon of mice after the colon barrier was damaged. Therefore, we confirmed the translocation of *E. coli* from the gut to the pituitary in mice.

### 2.9 Pituitary Cell Gap Junction and NLRP3 Ubiquitination Degradation Regulated by DRD2

**FIGURE 9:**
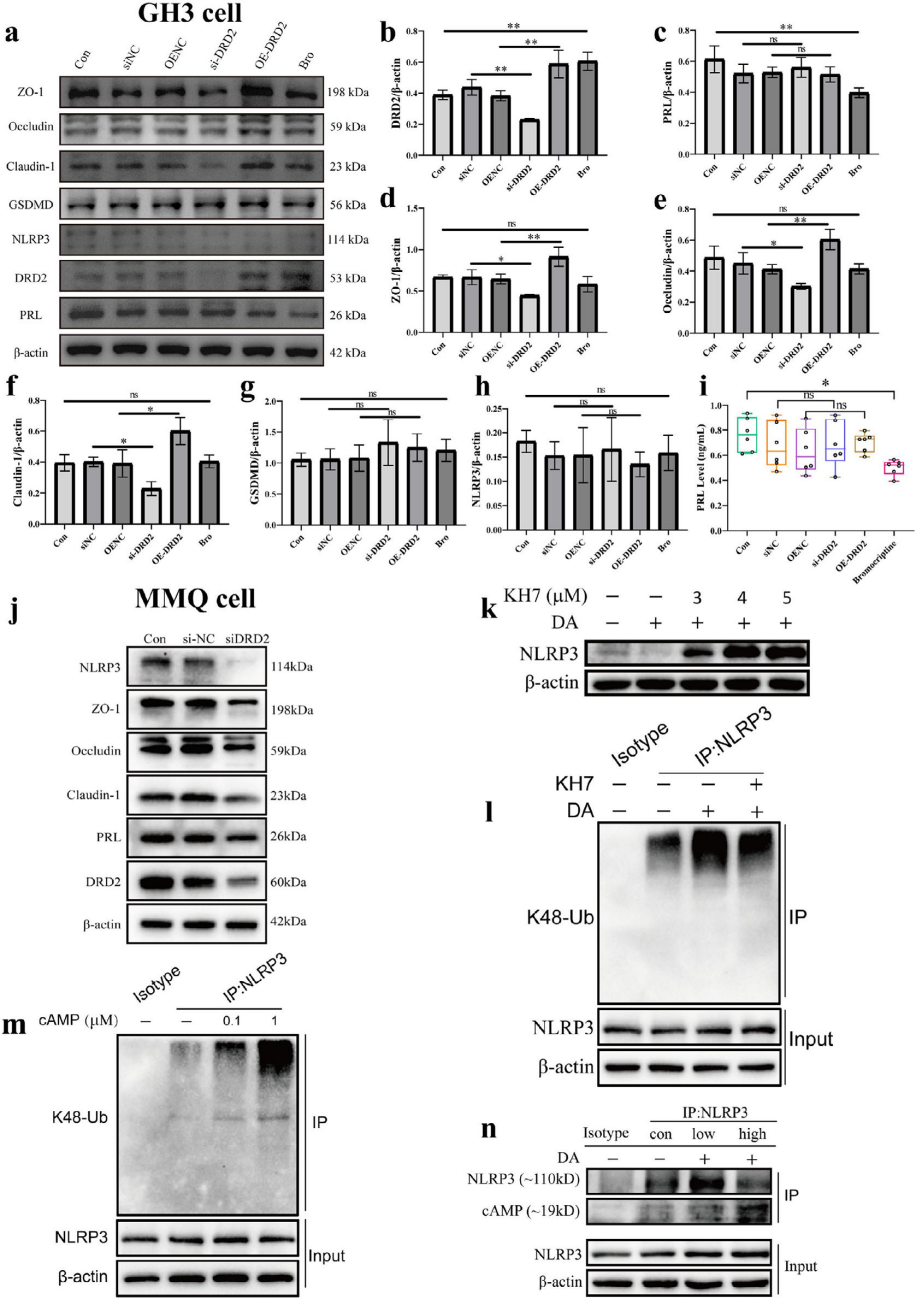
Pituitary cell gap junction and NLRP3 ubiquitination degradation regulated by DRD2. (a–h) Immunoblot analysis of cell gap junction proteins and PRL expressions of GH3 cell after DRD2 overexpressed or knockdown or treated with Br individually. n = 6. (i) Supernatant PRL level of GH3 cell after DRD2 overexpressed or knockdown or treated with Br individually by ELISA. n = 6. ^*^ *p* < 0.05, ^**^ *p* < 0.01, ^***^ *p* < 0.001, and ^****^ *p* < 0.0001. (j) Immunoblot analysis of cell gap junction proteins and PRL expressions of MMQ cell after DRD2 knockdown. n = 3. (k) Immunoblot analysis of NLRP3 and β‐actin in cell lysates from GH3 cells treated with different doses of KH7 before DA treatment. (l) GH3 cells were treated with KH7 before DA treatment. Immunoblot analysis of K48‐Ub protein from the cell lysates immunoprecipitated with anti‐NLRP3 antibody. (m) Lysates from GH3 cells were treated with different doses of cAMP. Immunoblot analysis of K48‐Ub protein from the cell lysates immunoprecipitated with anti‐NLRP3 antibody. (n) GH3 cells were treated with DA. Immunoblot analysis of cAMP and NLRP3 proteins from the cell lysates immunoprecipitated with anti‐NLRP3 antibody. Data were expressed as mean ± S.D. The one‐way ANOVA or Student's t‐test were used to perform statistical comparison by SPSS software. ^*^ *p* < 0.05, ^**^ *p* < 0.01, ^***^ *p* < 0.001, and ^****^ *p* < 0.0001.

To further confirm the effect of DRD2 on pituitary cell gap junction, DRD2 was overexp‐ ressed or knockdown or treated with agonist bromocriptine (Br) in GH3 cells or MMQ cells individually. The results revealed that the cell gap junction protein ZO‐1 (*p *< 0.05), Occludin (*p *< 0.05), Claudin‐1 (*p *< 0.05) expressions were all increased after DRD2 overexpressed while decreased after DRD2 knockdown in GH3 cell or MMQ cell lysates siginificantly (Figure 9a,d,e,f,j, *p *< 0.05), suggesting that DRD2 protein could regulate the cell gap junction effectively. However, supernatant PRL and PRL, NLRP3, and GSDMD proteins of lysates were all not changed whenever DRD2 overexpressed or knockdown in GH3 cells (Figure 9a,c,g–i, *p *> 0.05) while PRL and NLRP3 proteins of lysates were both changed after DRD2 knockdown in MMQ cells (Figure 9j, *p < *0.05). However, PRL level of cell supernatant or lysate were both decreased significantly after treatment with Br in GH3 cells (Figure 9a,i, *p *< 0.05), but with no effect on ZO‐1, Occludin, Claudin‐1, NLRP3, and GSDMD protein expressions (Figure 9a,c–e, *p *> 0.05). These results suggest that the pituitary cell gap junction was regulated by DRD2 significantly. Similarly, the regulating effect of DRD2 on cell gap junction of intestinal enterocyte were also investigated. The intestinal enterocytes were transfected with siRNA against DRD2, and tight junction proteins were assessed 24 hours post‐transfection. The results showed that ZO‐1, Occludin and Claudin‐1 protein expression levels were decreased significantly after DRD2 knockdown in intestinal enterocyte (Figure S5), indicating that DRD2 control the integrity of intestinal barrier, potentially facilitating the translocation of microorganisms from intestinal lumen into systemic circulation.

The DRD2 signaling cascade is capable of stimulating adenylate cyclase activity, which in turn, catalyzes the synthesis of cyclic adenosine monophosphate (cAMP). As a critical second messenger, cAMP is integral to a multitude of biological processes [28]. Recent literature has highlighted cAMP's inhibitory role in the activation of NLRP3 inflammasome [29]. DA and DRD1 signaling can also suppress NLRP3 inflammasome activation and identify an endogenous regulatory mechanism for NLRP3 inflammasome‐related inflammation [30]. In addition, significant inflammatory response was confirmed in the CNS of DRD2 knockout mice, suggesting that DA and its downstream signaling has an anti‐inflammatory function [31].In accord with this, the DA deficiency is closely associated with immune system abnormalities and CNS inflammation in Parkinson disease (PD) [32]. Therefore, we set out to elucidate the contribution of cAMP to the dampening of the inflammasome response triggered by dopamine (DA). The modulatory effect of DRD2 signaling pathway on NLRP3 ubiquitination was studied. Strikingly, the suppressive impact of DA on the NLRP3 inflammasome is counteracted by KH7, an inhibitor of adenylate cyclase, as depicted in Figure 9k. This observation underscores the cAMP‐dependent nature of the DA‐induced inhibition of the NLRP3 inflammasome. In alignment with this, the degradation and ubiquitination of NLRP3, induced by DA, are also impeded by KH7, as illustrated in Figure 9l. Moreover, cAMP has been shown to facilitate the ubiquitination of NLRP3 within lysates of pituitary tumor cells, as presented in Figure 9m. This suggests that elevated intracellular levels of cAMP might expedite the ubiquitination and subsequent degradation of NLRP3. Further corroborating these findings, immunoprecipitation of NLRP3 followed by detection with an anti‐cAMP antibody revealed bands corresponding to the molecular weight of NLRP3, as shown in Figure 9n. This indicates a potential direct interaction between NLRP3 and cAMP, potentially mediated through covalent bonding. Notably, treatment with DA markedly intensified the interaction between cAMP and NLRP3. Collectively, these results indicate that DA‐induced cAMP promotes NLRP3 ubiquitination and degradation in GH3 cells.

### 2.10 Clinical Relevance Studies

**FIGURE 10:**
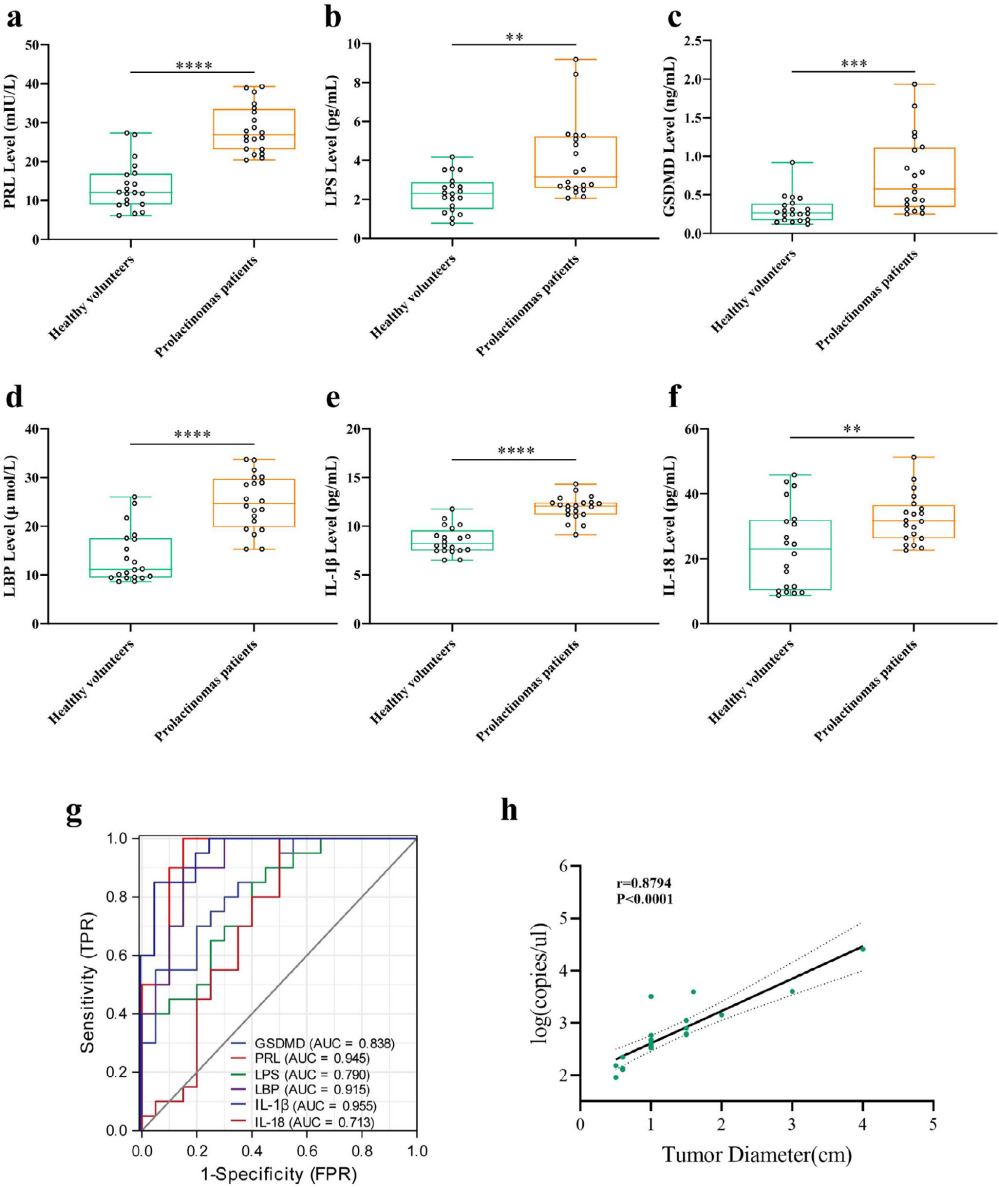
Clinical relevance studies. (a–f) Serum PRL (a), LPS (b), GSDMD (c), LBP (d), IL‐1β (e), and IL‐18 (f) concentrations in healthy volunteers and prolactinomas patients. n = 20. (g) ROC curves analysis for inflammatory genes in clinical sample data. (h) Correlation analysis between pituitary tumor diameter and contents of *Escherichia coli*. n = 20. Data were expressed as mean ± S.D. The one‐way ANOVA or Student's t‐test were used to perform statistical comparison by SPSS software. ^*^ *p* < 0.05, ^**^ *p* < 0.01, ^***^ *p* < 0.001, and ^****^ *p* < 0.0001.

At last, we investigated the clinical relevance. The blood of healthy volunteers (n = 20) and prolactinomas patients (n = 20) were collected. The serum PRL (*p *< 0.0001), LPS (*p *< 0.01), LBP (*p *< 0.0001), IL‐1β (*p *< 0.0001), IL‐18 (*p *< 0.01), and GSDMD (*p *< 0.001) levels of prolactinomas patients were all elevated significantly than those of healthy volunteers (Figure 10a–f). Receiver operating characteristic (ROC) curve analysis revealed that LBP, IL‐1β, PRL, GSDMD, LPS, and IL‐18 exhibit superior predictive potential for prolactinomas (area under the ROC curve: GSDMD: 0.838, PRL: 0.945, LPS: 0.790, LBP: 0.915, IL‐1β: 0.955, and IL‐18: 0.713), indicating the utility of these markers in prolactinomas diagnosis (Figure 10g). Thus, these serum inflammatory cytokines are elevated significantly in prolactinomas patients and may serve as potential biomarkers for pituitary adenoma. And the tumor diatemters of prolactinomas patients were positively correlated with contents of *Escherichia coli* significantly (*p *< 0.001, r = 0.8794, Figure 10h). More, human patients samples with functional prolactinomas and non‐functional pituitary tumors have been used to validate our findings. The results showed that pituitary PRL, NLRP3, GSDMD, HMGB1, IL‐1β, IL‐18 protein expressions of functional prolactinomas patients were increased significantly than those of non‐functional pituitary tumor patients, but pituitary DRD2, ZO‐1, Occludin, and Claudin‐1 expressions were decreased significantly (Figure S19).

## 3 Discussion

The pathogenesis of pituitary adenoma remains poorly clarified. Recently, the tumor cell microenvironment in the occurrence and progression of cancer is attracting increasing attention, including immune cells, stromal cells, cytokines, and microbe [43, 44]. It was reported that IL‐1 could act on the pituitary cells to regulate the release of ACTH, LH, GH, and TSH [45, 46, 47]. Our previous studies have confirmed that NLRP3 inflammasome activation mediated IL‐1β and IL‐18 promoted the formation of prolactinomas, and inhibition of NLRP3 or TLR4 all reversed the pathological process [17, 18].

In our usual understanding, the brain environment is recognized as sterile, as the BBB protects the central nervous system from infections or harmful substances [48]. Surprisingly, we have identified intracellular bacteria in the pituitary tumors. These live bacteria predominantly resided in the cytosol of pituitary prolactin‐producing tumor cells. Using metagenomic next‐generation sequencing and mass spectrometry techniques, we confirm that the bacterial species of pituitary tumor tissues is *Escherichia coli*. And more, in vivo tracing and immunofluorescence assay results showed that *Escherichia coli*. entered the pituitary tissue of mice treated with ES injection or heterozygous (DRD2 ±) or knockout (DRD2 −/−) mice after the blood pituitary barrier were destroyed. GSDMD integrate various inflammatory stimuli in brain endothelial cell (bECs), resulting in blood‐brain barrier (BBB) leakage and brain inflammation. *Escherichia coli*. from gut entered the pituitary tissue in mice through damaging BPB, which was reversed by GSDMD deficiency. Considering that intraperitoneal injection bypasses physiological gut barrier penetration, two ways of administration methods intragastric gavage or intrarectal instillation of fluorescent *Escherichia coli* were performed in DRD2(−/−) mice, respectively. The results showed that fluorescent *Escherichia coli* were both detected in the pituitary tissues of DRD2(−/−) mice whenever treated by intragastric gavage or intrarectal instillation of *Escherichia coli*. Therefore, our study claims that gut‐derived *E. coli* translocate to the pituitary. And serum‐to‐pituitary tracer assays by FITC‐dextran in DRD2(−/−) mice has been evaluated. The results showed that fluorescent FITC‐dextran was detected in the pituitary tissues of DRD2 (−/−) mice, suggesting that the blood‐brain barrier permeability was increased significantly in DRD2 (−/−) mice.

Functional barrier disruption in the gut is one of the three principal mechanisms promoting bacterial translocation from the intestinal lumen into the systemic circulation and extraintestinal location [49]. DRD2 was expressed both in the pituitary gland epithelium and in the gut. The formation of cellular junctions between PGE cells in the pituitary gland and between enterocytes in the lower gastrointestinal tract was impaired by the loss of DRD2, and this was associated with multiple intralesional bacteria in the degenerated pituitary gland of prolactinomas mice. We demonstrate that defective barrier function in the pituitary gland and colon led to translocation of intestinal *Escherichia coli* and inflammation in the pituitary gland, whereas removal of bacteria by germ‐free (GF) derivation, or systemic treatment with broad‐spectrum antibiotics successfully prevented the occurrence and progression of pituitary adenoma.

The tumor microbiome has been reported to drive the occurrence and progress of cancers. *Pseudomonas gingivalis* colonizes tumor tissue and activates NLRP3 inflammasome in the immune microenvironment, ultimately promotes the progression of colorectal cancer [50]. Intracellular pathogenic fungi promote pancreatic ducts by activating mannose‐binding lectin (MBL) to drive complement cascade reactions and promote the tumor growth of adenocarcinoma [51]. As an important natural immune response in the body, pyroptosis plays a key role in combating infection, and is closely related to the occurrence and metastasis of many cancers. It is a double‐edged sword for cancer, which can promote or inhibit the occurrence of tumors [52]. Cytokine release induced by GSDMD‐mediated pyroptosis such as IL‐1 and IL‐18, can promote abnormal proliferation and infiltration of tumors, leading to tumor occurrence and metastasis [53]. And more, HMGB1 released from pyroptotic cells induce tumor cell proliferation and PCNA expression, and participates in the tumorigenesis of various types of tumors through the ERK1/2 pathway [21, 22]. To confirm the role of bacteria in the pituitary adenoma, germ‐free mice were used and the differential signaling pathway of pituitary tissues between GF mice and SPF mice induced by ES were analysed by RNA sequencing. KEGG pathway analysis showed that NOD‐like receptor signaling pathway was enriched significantly. And pituitary NLRP3, Caspase‐11 and GSDMD protein expressions of GF mice were diminished significantly than those of SPF mice induced by ES. We found that the pathobiont *Escherichia coli* translocates from the gut into pituitary gland along with DRD2 loss and are phagocytosed by microglial cell.

Microglia are important immune cells in the central nervous system (CNS). Dysfunctions of gene‐deficient microglia contribute to the development and progression of multiple CNS diseases. Microglia replacement by nonself cells has been proposed to treat microglia‐associated disorders [54, 55, 56, 57]. To further confirm that microglia phagocytose bacteria and drive tumorigenesis, microglial depletion experiments were performed in DRD2(−/−) mice by intraperitoneally injection with microglia remover (PLX5622) for 10 days. The results revealed that the pituitary microglia cells were cleared mostly, then the pituitary PRL and MAPK (p‐P38/P38, p‐JNK/JNK, p‐ERK/ERK) expressions were decreased significantly in pituitary tissues of DRD2(−/−) mice after treatment with PLX5622 compared with DRD2(−/−) mice. Therefore, our study confirmed that *E. coli* drives the tumorigenesis of pituitary adenoma by activating microglia GSDMD/ HMGB1/MAPK pathway.

An age‐dependent BBB breakdown in the hippocampus, a region involved in learning and memory that is damaged early in Alzheimer's disease. The BBB breakdown was found in the hippocampus in the living human brain during normal aging [42]. It has been reported that age‐related loss of dopamine 2 receptor in the human brain [58, 59]. The age of most patients with pituitary adenoma are relatively old in the clinical, whether this disease is associated with age‐dependent BBB breakdown is unknown. In our study, the effect of age‐dependent BBB breakdow and intestinal epithelial barrier defects on prolactinomas was studied in elderly mice. The pituitary and colon DRD2 expression were both decreased significantly as same as cell gap junction protein ZO‐1, Occludin, and Claudin‐1, while PRL expression was increased remarkably in elderly mice. More, the damage of blood brain barrier were destroyed, and *Escherichia coli*. from gut entered into the pituitary tissue in twelve month old mice. And more, there were also *Escherichia coli*. in the cerebral cortex, hypothalamus, cerebellum, and midbrain tissues of twelve‐month old mice. The results suggest that the intestinal barrier and blood pituitary barrier are both damaged while DRD2 decrease in elderly mice, and bacteria from the gut translocate to pituitary gland resulting in the tumorigenesis as the age of mice increase.

Overall, our results reveal the loss of DRD2 lead to failure of both pituitary and colonic epithelial barriers, enabling *Escherichia coli*. to translocate from the gut to the pituitary gland where they activate microglial cells and drive adenoma formation. Moreover, these results also suggest antimicrobial agents, microglial depletion, or HMGB1 inhibitor ethyl pyruvate have the potential to cure pituitary adenoma. There are also some potential limitations of this study, such as the potential off‐target effects of antibiotics, and whether repairing intestine DRD2 expression could prevent *E. coli* translocation and tumorigenesis. These shortcomings would be studied further in future.

## 4 Experimental Section

### 4.1 Study Patients

Tumor tissue samples from 20 patients with nonfunctional pituitary adenoma and 21 patients with prolactinomas were obtained after surgical excision at Renmin Hospital of Wuhan University and Tongren Hospital affiliated to Wuhan University (the third hospital of Wuhan) between February 2019 and May 2023. Blood samples from 40 healthy volunteers and 40 patients with prolactinomas were collected at Renmin hospital of Wuhan University and Tongren Hospital affiliated to Wuhan University (the third hospital of Wuhan) between January 2019 and October 2025. All these patients did not receive antibiotic therapy in recent one month before surgical excision. The study was approved by the ethical committee of Tongren hospital affiliated to Wuhan University (the third hospital of Wuhan) and Renmin hospital of Wuhan University (ethical approval NO. WDRY2023‐K125). Informed consents were obtained from all the patients. Additionally, this study has been conducted according to the Code of Ethics of the World Medical Association (Declaration of Helsinki) for experiments involving humans.

### 4.2 Bacteria Culture

For isolation of bacteria, the tumor tissue pieces of human or mice prolactinomas samples (about 2 mg) were homogenized in 20 µL of PBS under sterile conditions. PBS was used to evaluate the environmental contaminants as tissue surrogate by the same workflow. The same sample were used to culture bacterial in Mueller‐Hinton agar or blood agar plate, respectively. For Mueller‐Hinton agar culture, 5 uL sample homogenate was plated on the agar with or without vancomycin (23.66 mg/mL) and imipenem (5.915 mg/mL) at 37°C aerobically in the incubator. For blood agar plate culture, 10 uL sample homogenate was plated on the agar with or without vancomycin (23.66 mg/mL) and imipenem (5.915 mg/mL) at 37°C aerobically in the incubator. The plates were incubated at 37°C for 24 hour in Mueller‐Hinton agar conditions or in blood agar plate conditions.

### 4.3 16S RNA FISH Assay

The bacterial colonization of *Escherichia coli* in human pituitary gland or mice pituitary gland or colon tissues was determined with GDP1073 16S rRNA gene probe(GCATAAGCG TCGCTGCCG) labeled with the fluorophore Cy5 by FISH. FFPE blocks were sectioned, deparaffinized and incubated by different chemical reagents. Probes were diluted and hybridized to the tissue overnight at 37°C. Samples were then stained with 1 ng/mL DAPI for 5 min, and mounted with the Antifade Mounting Medium. Images were obtained on an inverted epifluorescence microscope (NIKON ECLIPSE E100).

### 4.4 Immunohistochemistry Assays

The pituitary gland or tumor specimens were colleted and performed immunohistochemical staining according to our previous reported method [17, 18]. The slides were stained with anti‐LPS (1: 2000, Hycult Biotech) or anti‐ki67 (1: 1000, Servicebio) antibody. The stained tissues were imaged and analyzed by a microscope (Leica DFC450 C, Leica Microsystems Inc., Germany).

### 4.5 Immunofluorescence Assay

The human or mice pituitary gland and colon tissues were obtained and performed immunofluorescence assay according to our previous reported method [17, 18]. Primary antibody against antibodies GSDMD (1:2000, Abcam), CD31 (1:1000, servicebio), DRD2 (1:500, Affinity Biosciences), ZO‐1 (1:500, Affinity Biosciences), Occludin (1:200, Affinity Biosciences), CD68 (1:2000, Servicebio), CD3 (1:2000, Servicebio) were added. The fluorescence images of sample tissues were obtained under a microscope and analyzed using Leica Application Suite X Software (Leica DFC450 C, Leica Microsystems Inc., Germany).

### 4.6 Transmission Electron Microscopy Assay

Fresh human or mice pituitary gland and colon tissue were collected to reduce mechanical damage. Before sampling, petri dishes with fixative for IEM was prepared, and small tissue blocks were removed from the human or animal body and immediately put into petri dishes, and then cut into small size of 1mm^3^ in the fixative. The tissue blocks were transferred into an EP tube with fresh IEM fixative for further fixation, which was fixed at 4°C. Tissue blocks were washed on the ice box with pre‐cooled 0.1 m PB (pH 7.4) for three times, 10 min each. Dehydrate was performed as followed: 30% ethanol for 20 min at 4°C, 50%, 70%, 80%, 85%, 90%, and 100% ethanol for 20 min at −20°C, respectively. Resin penetration was performed by 100% ethanol and LR white resin. At 4°C, the pure LR white resin was first dropped into the embedding capsule, then the sample was put into the pure resin and the capsule cap was covered loosely at first. The resin blocks were cut to 70 nm thin on the ultra microtome, and the tissues were fished out onto the meshes nickel grids with formvar film. The nickel grids with tissues were stored at 4°C for standby application. Images are observed and acquired under TEM.

### 4.7 Microbial Metagenomic Next‐Generation Sequencing

Total microbia genomic DNA samples of human nonfunctional pituitary adenoma and prolactinomas samples were extracted, according to the manufacture's instructions. The purity and quality of extracted DNAs were determined by the NanoPhotometer and a Qubit 3.0. The 0.5 µg of genomic DNA was shattered, and the interrupted DNA was filtered using magnetic beads. The DNA was repaired and adaptor was added, purification was performed using the magnetic beads. The products were amplified and enriched by PCR. The DNA nanosphere was formed by rolling ring amplification technology. After library construction, the sequencing library was then sequenced on DNBSEQ‐T7 at Bioyi Biotechnology Co., Ltd. (Wuhan, China). Samples were sequenced on the platform to get image files and original data. The sequencing and analysis service were finished by Bioyi Biotechnology Co., Ltd. Wuhan, China. Clean data was used for species annotation by using kraken2. After that, bracken and KrakenTools were used for statistic and format conversion of the results. Species distribution results were presented using krona, and species diversity was analyzed using scripts written in python and R. The LEfSe(Linear discriminant analysis Effect Size) was used to find the species with significant differences in abundance between the groups (biomaker), and the two‐tailed test analysis was used to find the different species between the two groups. The data after quality control are assembled by megahit or spades, and biotool is used to rank and count the genomes. The contig of genes were annotated using kraken2, with non‐microbial data removed to obtain microbial genomes. The diversity of each sample was calculated for the assembled gene. Based on the classification and functional profiles of non‐redundant genes, LEfSe was used to determine differentially abundant taxa and functions across groups by the default paremeters.

### 4.8 Identification of Pituitary Microbial Species by Mass Spectrometry

The deionised water was added into a fine centrifuge tube, and two bacterial colonies of cultured pituitary glands were picked into the centrifuge tube and mixed well. Then 900 µL of anhydrous ethanol was added and mixed well. Later, it was centrifuged for 4 min, the supernatant was discarded and the precipitate was dried at 37°C for 5 min until there was no water stain on the surface of the precipitate. Then 10 µL of lysate 1 was added and mixed, then 10 µL of lysate 2 mixed well, and centrifuged for 4 min. Then 1 µL of supernatant was put onto the sample target and dried, and 1 µL of matrix solution was added to cover the sample spot and dry. At last, the sample was put into the fully automated microbial mass spectrometry detection system (Autof ms1000) for the identification of microbial species.

### 4.9 Animals

Female C57BL/6 mice (weighing 20–22 g, 1 months old) or female C57BL/6 mice (weighing 30–35 g, 12 months old) were purchased from Henan SKBES Biotechnology Co., Ltd (Anyang, PR China), which were housed and bred in specific pathogen‐free grade cages and provided with autoclaved food and water. GSDMD‐deficient (GSDMD−/−) mice were purchased from GemPharmatech Co., Ltd (Nanjing, China), DRD2‐deficient (DRD2± or DRD2−/−) mice were purchased from Cyagen Biosciences Inc (Suzhou, China), which were both housed and bred in specific pathogen‐free grade cages and provided with autoclaved food and water. Female C57BL/6 germ‐free mice (weighing 15–30 g) were purchased from Experimental Animal Center of Huazhong Agricultural University (Wuhan, PR China), which were housed and bred in sterile grade cage and provided with sterile food and water. All animal experiments were approved by the Ethics Committee of Tongren Hospital Affiliated to Wuhan University (The Third Hospital of Wuhan) (ethical approval NO. SY2022‐055). All animal experiments have complied with the Animal Research: Reporting of In Vivo Experiments guidelines and conformed to the National Institutes of Health Guide for the Care and Use of Laboratory Animals (NIH Publications No. 8023, revised 1978).

### 4.10 Prolactinomas Animal Model and Groups

The estradiol‐induced mice are a stable and reliable model of prolactinomas [17, 19, 20]. This prolactinomas animal model was established by intraperitoneally injecting estradiol oil in C57BL/6 mice (1 mg/1 mL of concentration, 0.1 mL for dosage each mice once) once every 4 days for 40 days. The mice were separated into four groups (n = 10 for each group): control, estradiol (ES)‐induced prolactinomas, germ‐free (GF) mice, and germ‐free mice induced ES groups. All mice were intraperitoneally injected with estradiol oil, except for control and germ‐free group mice, which were injected with 1 mL of castor oil for each mice.

Another set of female C57BL/6 mice were separated into four groups (n = 10 for each group): control, estradiol‐induced prolactinomas model, model plus one kind of antibiotic combination treatment (ATB1): vancomycin (47.32 mg/mL), Imipenem/ Cilastatin Sodium (11.83 mg/mL), neomycin(1 mg/mL), and amphotericin(1.66 mg/mL)), and model plus another kind of antibiotic combination treatment (ATB2): doxycycline (2.38 mg/mL), clarithromycin (11.85 mg/mL), and azithromycin (5.91 mg/mL)). All mice were intraperitoneally injected with estradiol oil (1 mg/1 mL of concentration, 0.1 mL for dosage each mice once) once every 5 days for 50 days, except the control group. The control group mice were injected with 1 mL of castor oil once. Two kinds of antibiotic combinations were given intragastrically to model mice, respectively, once every two days for 50 days.

The third group of female C57BL/6 mice were divided into three groups (6 mice in each group): normal control group, *Escherichia coli*‐treated group, and ES plus *Escherichia coli* ‐treated group. The *Escherichia coli*‐treated group mice was injected with one intraperitoneal injection of castor oil (1 mL), and then each mice was also injected with one injection of *Escherichia coli* (20 µL/1×10^10^), and one intraperitoneal injection of castor oil (1 mL) in the normal control group. The ES plus *Escherichia coli*‐treated group mice was injected intraperitoneally with estradiol oil (concentration of 1 mg/1 mL, 0.1 mL per mice per dose) once every 4 days for 40 consecutive days, in addition to one injection of *Escherichia coli* labeled with fluorescent (20 µL/1×10^10^) per mice intraperitoneally.

The fourth group of female mice were divided into four groups (six mice per group): normal control group, estradiol‐induced model group, GSDMD (−/−) control mice, GSDMD (−/−) plus estradiol group. Except for the normal control and GSDMD (−/−) control mice, all other mice were injected intraperitoneally with estradiol oil (concentration of 1 mg/1 mL, 0.1 mL per mice per dose) once every 4 days for 40 consecutive days.

The fifth group of female mice were divided into two groups (6 mice per group): GSDMD (−/−) control mice, GSDMD (−/−) plus estradiol group. These mice were treated with one injection of *Escherichia coli* labeled with fluorescent (20 µL/1×10^10^) per mice intraperitoneally.

The sixth group of female DRD2 (±) or DRD2 (−/−) mice were divided into two groups (six mice in each group), and each mice was injected with *Escherichia coli* labeled with fluorescent (20 µL/1×10^10^) per mice intraperitoneally.

The seventh group of female C57BL/6 mice was divided into two groups (ten mice per group): one group mice were 1 month old; the other group mice were 12 months old. Three mice of each group were randomly injected with *Escherichia coli* labeled with fluorescent (20 µL/1 ×10^10^) intraperitoneally.

The eighth group of female C57BL/6 mice (weighing 20–22 g) were separated into three groups (six mice in each group): control group, DRD2(−/−) mice group, DRD2(−/−) mice plus antibiotic treatment (aztreonam, 100 mg/kg) group. Aztreonam were given intragastrically in mice once a day for 10 days.

The ninth group of female C57BL/6 mice (weighing 20–22 g) were separated into three groups (six mice in each group): control group, DRD2(−/−) mice group, DRD2(−/−) mice plus ethyl pyruvate treatment (100 mg/kg) group. The ethyl pyruvate solution were injected intraperiton‐ eally in mice once a day for 10 days.

The tenth group of female C57BL/6 mice (weighing 20–22 g) were separated into two groups (five mice in each group): control group, DRD2(−/−) mice group. Intestinal permeability was evaluated with ffuorescein isothiocyanate (FITC)‐Dextran 4000 test. Mice were fasted for 4 h and then FITC‐dextran 4000 (600 mg/kg body wt) was administered by gavage. Four hours after gavage, whole blood was collected, and serum was isolated. The analysis for the ffuorescence intensity of FITC‐dextran 4000 in plasm was carried out with a multimode plate reader (excitation, 490 nm; emission, 520 nm). Standard curves for calculating the FITC‐dextran concentration were obtained by diluting FITC‐dextran in PBS.

The eleventh group of female C57BL/6 mice (weighing 20–22 g) were separated into four groups (three mice in each group): one control group, DRD2(−/−) mice group plus intragastric gavageof fluorescently labeled *Escherichia coli*, another control group, DRD2(−/−) mice group plus intrarectal instillation of fluorescently labeled *Escherichia coli*. The fluorescence intensity of *Escherichia coli* in the pituitary gland of mice was assessed after two weeks.

The twelfth group of female C57BL/6 mice (weighing 20–22 g) were separated into three groups (three mice in each group): control group, DRD2(−/−) mice group, DRD2(−/−) mice group plus PLX5622. Mice were intraperitoneally injected once daily with 0.65% PLX5622 suspended in 0.01 m PBS containing 5% dimethyl sulfoxide and 20% Kolliphor RH40 or an equal volume of vehicle for ten consecutive days.

### 4.11 RNA Sequencing

Total RNA was extracted from pituitary gland tissues using Trizol (Invitrogen, Carlsbad, CA, USA). Total RNA was extracted from tissues according to the instructions. The tissues were ground to powder with liquid nitrogen in 2 mL tubes. Approximately 60 mg of tissues were ground to powder with liquid nitrogen into chloroform/isoamyl alcohol (24: 1). The upper aqueous phase with residual RNA was transferred to a new tube by centrifugation, and an equal volume of isopropanol was added to it After centrifugation and discarding the supernatant, the RNA was washed with 75% ethanol and allowed to air dry. Finally, DEPC water was added to dissolve the RNA. Then, the Nano Drop and Agilent 2100 Bioanalyzer (Thermo Fisher Scientific, Massachusetts, USA) were used. Oligo(dT)‐attached magnetic beads were used to purify mRNA. Purified mRNA was fragmented into small pieces with fragment buffer at the appropriate temperature. Then First‐strand cDNA was generated using random hexamer‐primed reverse transcription, followed by a second‐strand cDNA synthesis. Afterward, A‐Tailing Mix and RNA Index Adapters were added by incubating to end repair. The cDNA fragments obtained from the previous step were amplified by PCR, and products were purified by Ampure XP Beads, then dissolved in EB solution. The product was validated on the Agilent Technologies 2100 bioanalyzer for quality control. The double stranded PCR products from the previous step were heated denatured and circularized by the splint oligo sequence to get the final library. The single strand circle DNA (ssCir DNA) was formatted as the final library. The final library was amplified with phi29 to make DNA nanoball (DNB) that had more than 300 copies of one molecular, DNBs were loaded into the patterned nanoarray, and single end 50 bases reads were generated on MGISEQ2000 platform (BGI‐Shenzhen, China).

### 4.12 Cell Culture and Treatment

The GH3 and MMQ cell line was obtained from the Beijing Beina Chuanglian Biotechnology Research Institute (Beijing, China). GSDMD‐stably knockdown GH3 cells (Admindin) and control cells (adGFP) were established by Wuhan Yingji Technology Co., Ltd (Wuhan, China). These cells were maintained in MEM media that contained 10% fetal bovine serum (FBS) at 37°C under normoxic conditions (95% air/ 5% CO_2_). The *Escherichia coli* (10 µL, 1 × 10^8^/mL, Lot No. ALG1173A) was electroporated into untreated or GSDMD knockdown‐treated GH3 cells by the electroporator (Thermo Invitrogen). The GH3 cell morphology were observed by electronic microscopes, and the cell proliferation were determined by CCK8 at 3 or 6 h. The PRL and GSDMD‐mediated pyroptosis pathway proteins of GH3 cells were determined by Western blot. And PRL, LBP, IL‐1β and IL‐18 levels of GH3 cell supernatants were measured by ELISA.

GH3 cells were treated with different concentrations of KH7 (3, 4, and 5 µm Lot: 116236, MedChemExpress) for 1 h, and then treated with DA (0.15 µm, Lot: 312294, MedChemExpress) for 12 h. Cells were collected for Western blot analysis; GH3 cells were treated with KH7 (4 µm) for 1 h and then treated with DA (0.15 µm) for 12 h. Cells were collected for Western blot analysis. GH3 cells were treated with different concentrations of cAMP (0.1, 1 µm, Lot: 151961, MedChemExpress) for 12 h. Cells were collected for Western blot analysis. An plasmid and siRNA sequence against DRD2 and DRD2 plasmid sequence was designed and synthesized by Wuhan GeneCreate Biological Engineering Co., LTD. (Wuhan, China). The sequences were as follows: Forward primer: GGAAGCG GGUCAACA CCAATT; Reverse primer: UUGGUG UUGACCCGCUUCCTT. A total of 2 × 10^5^ GH3 or MMQ cells per well were seeded into a six‐well plate and then transfected with the DRD2 siRNA sequences or DRD2 plasmid sequence or Bromo‐ criptine (5 µg/mL, Lot:385932, MeChemExpress) separately using Lipofectamine 2000 (Invitrogen; Thermo Fisher Scientific, Inc.) according to the manufacturer's protocol. The protein was extracted 48 h later. And PRL levels of GH3 cell supernatants were measured by ELISA.

The intestinal enterocytes were obtained from Qingqi (Shanghai) Biotechnology Development Co., Ltd, and transfected with siRNA against DRD2 using Lipofectamine 2000 (Thermo Fisher Scientific, USA), and the tight junction proteins ZO‐1, Occludin and Claudin‐1 of intestinal enterocyte were assessed 24 hours post‐transfection by Western blot. The sequences of the siRNA oligonucleotides were as follows: Negative control (NC) siRNA: Sense: 5′‐ UUCUCCGAACGUGUCACGUTT‐3′, Antisense: 5′‐ ACGUGACA CGUUCGGAGAATT ‐3′; DRD2 siRNA: Sense: 5′‐CCACUACA ACUACUAUGCCAUTT‐3′, Antisense: 5′‐AUGGCAUAGUAGUUGUAGUGGTT‐3′.

### 4.13 Cell Viability Assay

Cell Counting Kit‐8 (CCK‐8) assay was used to measure the GH3 cell viability. Log‐phase GH3 cells were collected and the cell density was adjusted to 1 × 10^5^ cells /mL according to the kit instructions (Wuhan Servicebio Technology Co., Ltd.). Then each well in a 96‐well plate was added with 100 µL of the cell suspension and ten microlitres of the CCK‐8 stock solution. After that, the OD values were determined at 450 nm by absorbance microplate reader at 0, 3, 6, and 12 h (Bio‐Rad imark, USA). The cell viability was calculated by OD values. A total of 5 × 10^3^ GH3 cells were cultured in 96‐well plates with 100 µL medium, infected with *E. coli* bacterial suspension (MOI =  1, 10, and 100) in 100 µL medium, and incubated at 37°C for 3, 6, 12, and 24 h. After treatment with 20 µL CCK‐8 reagent for 1 h, cell viability was quantified using a microplate reader. GH3 cell was cultured in 6‐well plates (5 × 10^4^ cells/well) for 24 h, infected with *E. coli* bacterial suspension (MOI =  1, 10, and 100), and incubated at 37°C for 3, 6, 12, and 24 h. Images were captured using a digital camera (Olympus IX37) with cellSens Dimension software (v2.3). PRL and MAPK protein expression of GH3 cell were determined after infected with *E. coli* (MOI =  1, 10, or 100) at 3 h by Western blot.

### 4.14 Enzyme‐Linked Immunosorbent Assay (ELISA)

The human or mice serum were obtained and then isolated by centrifugation immediately. For the PRL, LPS, LBP, IL‐1β or IL‐18 levels of human, mice serum or cell supernatants, ELISA was used according to our previous reported methods [17, 18]. The ELISA kits were all purchased from Ruixin Biotechnology Co., Ltd (Quanzhou, China), and the batch numbers were as followed: human PRL: No.RX106036H, human LPS: No.RX104820H, human LBP: No.RX104819H, human IL‐1β: No.RX106152H, human IL‐18: No.RX106154H; human GSDMD:No.RX100440H; mice PRL: No.RX202993M, mice LPS: No.RX202425M, mice LBP: No.RX202132M, mice IL‐1β: No.RX203063M; mice GSDMD:No.RX200648M; rat PRL: No.RX302794R, rat LPS: No.RX303098R, rat LBP: No.RX302076R, rat IL‐1β: No.RX302869R, rat IL‐18: No.RX302871R.

### 4.15 Fluorescence Quantitative PCR(FQ‐PCR)

The genomic DNA was extracted from human pituitary prolactin‐producing tumor tissues. Then agarose gel electrophoresis was carried out, and bacterial liquid was identified by PCR. Later, microbial sequencing was used to clarity microbial species. At last, the microbial quantification of human pituitary adenoma or mice pituitary tissue and feces was performed by fluorescence quantitative PCR. The primer information of *Escherichia coli* is as followed. TTGAGTCTCGTAGAGGGGGGTA (forward primer), TCAAGGGCAC AACCTCCAAGT (reverse primer).

### 4.16 Evans Blue Dye

Mice were injected with 2% Evans blue in the tail vein, and 2 h later, the mice were deeply anesthetized and cardiac perfusion was performed with saline, then the skulls were carefully peeled off. The brain tissue and pituitary gland were delicately dissected and photographed using iphone XR cellphone camera.

### 4.17 Magnetic Resonance Imaging

MRI experiments of pituitary gland were performed on a 7.0T vertical bore Bruker Biospec 70/30 scanner (BrukerBioSpin MRI GmbH, Rheinstetten, Germany). The parameters used in the scans were optimized for gray‐white matter contras. Based on reference multislice RARE scans (Sagittal), with TR = 2500 ms, echo train length = 8, TEeff (Echo Time) = 33 ms, field‐of‐view (FOV, Rectangle) = 18 × 18 mm and matrix size = 256 × 256 × 12, voxel size was 0.0703125 × 0.0703125 ×0.5 mm. Total imaging time was 32 min.

### 4.18 In Vivo Imaging of *Escherichia coli*

The *E. coli* bacterial solution was streaked on LB medium and incubated overnight. Single colonies were picked in LB medium and cultured overnight at 37°C for activation. Transferred to fresh LB medium at 1/100 of the inoculum volume, cultured to mid‐late logarithmic growth (OD600 = 0.3–0.6) and then cooled in an ice bath for 30 min, centrifuged for 5 min, the supernatant was discarded, and drained on sterile filter paper; resuspended the cells with half volume of the pre‐cooled cultures in 0.1 mol/L CaCl_2_ solution, and incubated in an ice bath for 30 min. centrifuged, and the bacterium was re‐collected; resuspended with 1/20 volume of the initial cultures in 0.1 mol/L CaCl_2_ solution. 1/20 initial culture volume of CaCl_2_ solution (85 mmol/L CaCl_2_ and 15% glycerol) to resuspend the cells and obtain the receptor cells ‐80°C for storage. Add 1 µL of GFP plasmid (promoter T7) to 100 µL of the above receptor cells, incubate on ice for 30 min, heat‐excite at 42°C for 90 s, and incubate on ice for 2 min. Add 500 µL of liquid LB medium, recover at 37°C and 200 rpm shaker for 1 h, centrifugate at 10 000 g for 1 min, and discard part of the supernatant. The remaining about 100 µL of supernatant was spread on Kana‐resistant plates and incubated at 37°C overnight. After overnight incubation of the plate, pick the strain that showed green colour under natural light, put it into 37°C for overnight incubation, extract the plasmid on the next day, sequence it, and pick the plasmid with the correct sequencing results. The *E. coli* bacterial solution prepared as described above was injected intraperitoneally into mice (20 µL/1×10^10^), and fluorescence imaging was performed at different time points at emission wavelength of 540 nm and excitation wavelength of 475 nm.

### 4.19 Western Blot

The tissue protein of human or mice pituitary gland samples were lysed with protease inhibitors. The protein concentrations of mice or human tissues or cell lysates were determined by Bradford protein assay. Proteins were separated by SDS‐PAGE according to our previous reported methods [17, 18]. The primary antibodies were used as follows: NLRP3 antibody from mouse (AdipoGen Life Sciences, San Diego, USA, 1:6000), Caspase‐11 antibody from rabbit (Affinity Biosciences, 1:3000), IL‐1β antibody from rabbit (Proteintech Group, Inc., 1:3000), IL‐18 antibody from rabbit (Affinity Biosciences, 1:3000), GSDMD antibody from goat (abcam, 1:6000), prolactin (PRL) antibody from rabbit (Affinity Biosciences, 1:3000), DRD2 antibody from rabbit (Affinity Biosciences, 1:3000), LBP antibody from rabbit (Affinity Biosciences, 1:3000), ZO‐1 antibody from rabbit (Affinity Biosciences, 1:3000), Claudin‐1 antibody from rabbit (Affinity Biosciences, 1:4000), Occludin antibody from rabbit (Affinity Biosciences, 1:3000), cAMP antibody from rabbit (Affinity Biosciences, 1:3000), S100A8 antibody from rabbit (Affinity Biosciences, 1:3000), S100A9 antibody from rabbit (Affinity Biosciences, 1:3000), HMGB1 antibody from rabbit (Affinity Biosciences, 1:3000), JNK antibody from rabbit (Affinity Biosciences, 1:3000), p‐JNK antibody from rabbit (Affinity Biosciences, 1:3000), ERK antibody from rabbit (Affinity Biosciences, 1:3000), p‐ERK antibody from rabbit (Affinity Biosciences, 1:3000), P38 antibody from rabbit (Affinity Biosciences, 1:3000), p‐P38 antibody from rabbit (Affinity Biosciences, 1:3000) and β‐actin antibody from rabbit (Affinity Biosciences, 1:6000). All protein expression of samples were quantified by Quantity OneV 4.6.6. The band intensity was normalized to the β‐actin band.

### 4.20 Statistical Analyses

All quantitative data in the figures were expressed as mean ± S.D. The one‐way ANOVA (Multiple Comparison) or Student's t‐test (Pairwise Comparison) were used to perform statistical comparison by SPSS software (version 20.0). Asterisks indicate the statistical significance level (^*^ *p* < 0.05, ^**^ *p* < 0.01, ^***^ *p* < 0.001, and ^****^ *p* < 0.0001). *p* < 0.05 was considered statistically significant.

## Author Contributions

Conceptualization, X.W., L.Q.W., X.D.H., M.P.; methodology, X.X. and Y. Z.; resources, S.G.D., Q.Y.Z., and J.H.M.; experiments and image analysis, X.J.S., L.M., Q.W., and L.L.L.; writing – review & editing, H.F.Z., Y.F.W.; supervision, L.M., and J.H.W. All authors have read and approved the final manuscript.

## Conflicts of Interest

The authors declare no conflict of interest.

## Supporting information




**Supporting File**: advs73545‐sup‐0001‐SuppMat.doc.

## Data Availability

The data that support the findings of this study are available from the corresponding author upon reasonable request.
