# Supplementary material for: DRD2 Deficiency Underlies Pituitary Adenoma Dependent on Escherichia coli Translocation from the Gut
Source: Adv Sci (Weinh). 2026 Feb 8;13(24):e04247. doi: 10.1002/advs.202504247 (PMC13116083; doi:10.1002/advs.202504247)
Supplement: Supplementary file 1 — Supporting File: advs73545‐sup‐0001‐SuppMat.doc. [file ADVS-13-e04247-s001.doc]

**SUPPORTING INFORMATION**

**DRD2 Deficiency Underlies Pituitary Adenoma Dependent on *Escherichia coli* Translocation from the Gut**

**Xian-jun Su^1,2,3†^, Li Ma^1†^, Xi Xiong^4†^, Jun-hua Meng^1†^, Qi Wu^1†^, Yu Zhang^5†^, Shu-guang Dong^6†^, Yue-fei Wang^7^, Jin-hu Wu^1^, Qing-yuan Zeng^1^, Hong-feng Zhang^8*^, Li-li Li^9*^, Liang Meng^7*^, Min Peng^10*^, Xiao-dong Huang^11*^, Li-quan Wu^12*^, Xiong Wang^1,13*^**

^1^Department of Pharmacy, Tongren hospital affiliated to Wuhan University (the third hospital of Wuhan), Wuhan 430060, China

^2^College of Pharmacy, Wuhan University of Science and Technology, Wuhan 430081, China

^3^Department of Nuclear Medicine, Tongji Hospital affiliated to Tongji Medical College, Huazhong University of Science and Technology, Wuhan, 430030, China

^4^Department of Urology surgery, Tongren hospital affiliated to Wuhan University (the third hospital of Wuhan), Wuhan 430060, China

^5^Department of Pharmacy, Pulmonary Hospital of Wuhan, Wuhan 430030, China

^6^Department of Cardiology, Tongren hospital affiliated to Wuhan University (the third hospital of Wuhan), Wuhan 430060, China

^7^Department of Neurosurgery, Tongren hospital affiliated to Wuhan University (the third hospital of Wuhan), Wuhan 430060, China

^8^Department of Pathology, the central hospital of Wuhan, Tongji medical college, Huazhong University of Science and Technology, Wuhan 430014, China

^9^Central Laboratory, Renmin hospital of Wuhan University, Wuhan 430060, China

^10^Department of Oncology, Renmin Hospital of Wuhan University, Wuhan 430060, China

^11^Department of Gastroenterology, Tongren hospital affiliated to Wuhan University (the third hospital of Wuhan), Wuhan 430060, China

^12^Department of Neurosurgery, Renmin hospital of Wuhan University, Wuhan 430060, China

**^†^**These authors contributed equally.

^13^Lead contact

^*^Corresponding author. Email: [zhf152@163.com](mailto:zhf152@163.com) (H.-F.Z.), [26061047@qq.com](mailto:26061047@qq.com) (L.-L.L.), [meng liang_whu@163.com](mailto:meng liang_whu@163.com) (L.M.), [mpeng320@whu.edu.cn](mailto:mpeng320@whu.edu.cn) (M.P.), [13886190549@139.com](mailto:13886190549@139.com) (X.-D.H.), [wuliquan@whu.edu.cn](mailto:wuliquan@whu.edu.cn) (L.-Q.W.), [wangxiong-133494@163.com](mailto:wangxiong-133494@163.com) (X.W.)

**SUPPORTING FIGURES**

**Figure S1.** Effect of aztreonam or ethyl pyruvate on pituitary PRL and inflammatory factors

in DRD2(-/-) prolactinomas mice.

(a) Immunoblot analysis of pituitary PRL and inflammatory factors in DRD2(-/-) prolactinomas mice treated with aztreonam. n=6.

(b) Immunoblot analysis of pituitary PRL and inflammatory factors in DRD2(-/-) prolactinomas mice treated with ethyl pyruvate. n=6. Data were expressed as mean ± S.D. The one-way ANOVA or Student’s t-test were used to perform statistical comparison by SPSS software. ^*^*p* < 0.05, ^**^*p* < 0.01, ^***^*p* < 0.001, and ^****^*p* < 0.0001.

**Figure S2.** *Escherichia coli* directly induces tumorigenesis of pituitary gland.

(a) Cell proliferation of GH3 cells were determined after infected with *Escherichia coli* (MOI = 1, 10, or 100) at 3 h, 6 h, 12 h, or 24 h by CCK8.

(b) Cell morphology of GH3 cells after infected with *Escherichia coli* (MOI = 1, 10, or 100) at 3 h, 6 h, 12 h, or 24 h by electronic microscopes (magnification 10× ).

(c-d) PRL and MAPK protein expression of GH3 cell were determined after infected with *Escherichia coli* (MOI = 1, 10, or 100) at 3 h by Western blot. n=3. Data were expressed as mean ± S.D. The one-way ANOVA or Student’s t-test were used to perform statistical comparison by SPSS software. ^*^*p* < 0.05, ^**^*p* < 0.01, ^***^*p* < 0.001, and ^****^*p* < 0.0001.

**Figure S3.** Damage of blood pituitary barrier in DRD2 (+/-) or DRD2(-/-) mice

(a) BPB defect change of pituitary gland in WT, DRD2 (+/-) or DRD2(-/-) mice by observing evans blue color. Left: brain tissue, right: pituitary gland

(b) *In vitro* tracing of *Escherichia coli.* labeled with fluorescent in DRD2 (+/-) or DRD2(-/-) mice. n=6.

(c) Pituitary *Escherichia coli.* determination by immunofluorescence assay in DRD2 (+/-) or DRD2(-/-) mice. n=6.

**Figure S4.** *Escherichia coli.* distribution in the colon of mice.

(a) Colon *Escherichia coli.* distribution in mice treated with intraperitoneal injection of

ES by FISH with *E. coli*-specific probe.

(b) Colon *Escherichia coli.* distribution in mice treated with intraperitoneal injection of

*Escherichia coli.* or ES plus *Escherichia coli.* by immunofluorescence assay.

Control: control mice, ES: estradiol-treated mice, Ecoli: mice treated with intraperitoneal

injection of *Escherichia coli.*, Ecoli+ES: mice treated with intraperitoneal injection of ES plus *Escherichia coli.*

**Figure S5.** Cell gap junction of intestinal enterocyte regulated by DRD2.

(a) Immunoblot results of tight junction proteins of intestinal enterocyte after DRD2 knockdown.

(b-d) Immunoblot analysis of tight junction proteins of intestinal enterocyte after DRD2 knockdown. n=3. Data were expressed as mean ± S.D. The one-way ANOVA or Student’s t-test were used to perform statistical comparison by SPSS software. ^*^*p* < 0.05, ^**^*p* < 0.01, ^***^*p* < 0.001, and ^****^*p* < 0.0001.

**Outer** **blood pituitary barrier defects with DRD2 decrease in pituitary gland of prolactinomas mice**

The blood-brain barrier (BBB) is formed by an interdependent network of brain capillary endothelial cells and perivascular cells, which is a strict regulation of paracellular permeability due to the presence of junctional complexes (tight, adherens and gap junctions) between the endothelial cells and alterations in junction assembly significantly affect barrier permeability

.^[25,26,27]^ Outer blood pituitary barrier (BPB) of mice was analysed by investigating the cell gap junction proteins. The results showed that the BPB of pituitary gland in mice became diffusely tightly connected, with damaged endothelial cells and no tissue structure gradually during the 40 days estradiol induction process by TEM analysis (Fig. S6a,b). Furthermore, the cell gap junction protein ZO-1, Occludin, and Claudin-1 expressions of pituitary gland in mice were gradually decreased during the 40 day-ES induction process (Fig. S6e,f,g,h, Figure S7b, all *p*<0.05). And pituitary DRD2 was mainly located at epithelium, and its expression decreased gradually during the 40 days-ES induction process by immunofluorescence assay (Figure S7a). Similarly, the BPB of pituitary gland in DRD2 (+/-) or DRD2 (-/-) mice both became diffusely tightly connected, with damaged endothelial cells and no tissue structure by TEM analysis (Fig. S6c,d). And the cell gap junction proteins ZO-1, Occludin, and Claudin-1 expressions of pituitary gland in DRD2 (+/-) or DRD2 (-/-) were all decreased significantly than those of WT mice (Fig. S6i,j,k,l, all *p*<0.01). These results further confirm the outer blood pituitary barrier defects with DRD2 decrease in pituitary gland of prolactinomas mice.

**Figure S6.** Outer blood pituitary barrier defects with DRD2 decrease in pituitary gland of prolactinomas mice

(a) BPB structure change of pituitary gland in mice during the 40 days-ES induction process by TEM analysis. TJ: tight junction

(b) Tight junction width of BPB of pituitary gland in mice during the 40 days-ES induction process. n=3.

(c) BPB structure change of pituitary gland in DRD2 (+/-) or DRD2 (-/-) mice by TEM analysis.

(d) Tight junction width of BPB of pituitary gland in DRD2 (+/-) or DRD2 (-/-) mice. n=3.

(e-h) Cell gap junction protein expressions of pituitary gland in mice during the 40 days-ES induction process by Western blot. n=3.

(i-l) Cell gap junction protein expressions of pituitary gland in DRD2 (+/-) or DRD2 (-/-) mice by Western blot. n=3. Data were expressed as mean ± S.D. The one-way ANOVA or Student’s t-test were used to perform statistical comparison by SPSS software. ^*^*p* < 0.05, ^**^*p* < 0.01, ^***^*p* < 0.001, and ^****^*p* < 0.0001.

**Figure S7.** Pituitary DRD2 and cell gap junction expression in prolactinomas mice

(a) The location and expression of pituitary DRD2 in mice induced by ES induction at different time points by immunofluorescence assay.

(b) The location and expression of pituitary cell gap junction protein Occludin and ZO-1 in mice induced by ES induction at different time points by immunofluorescence assay.

**Intestinal epithelial barrier defects with DRD2 decrease in the colon of prolactinomas mice**

Considering *Escherichia coli* live the intestine, whether the intestinal epithelial barrier was damaged in the colon of prolactinomas mice was studied. The results showed that the colonic microvilli became sparse, with damaged epithelium and widened cell gaps gradually during the 40 days-estradiol induction process in mice by TEM (Fig. S8a,b). Futhermore, the cell gap junction protein ZO-1, Occludin, and Claudin-1 expressions of colon were decreased while DRD2 diminished gradually during the 40 days-ES induction process (Fig. S8e,f,g,h,i, Figure S9b, Figure S10, all *p*<0.05). Similarly, the colonic microvilli became sparse, with damaged epithelium and widened cell gaps in DRD2 (+/-) or DRD2 (-/-) mice by TEM (Fig. S8c,d). And the cell gap junction protein ZO-1, Occludin, and Claudin-1 expressions of colon were all decreased in DRD2 (+/-) or DRD2 (-/-) mice (Fig. S8j,k,l,m,n, all *p*<0.05). Interestingly, colon DRD2 was mainly located at intestinal epithelium, and its expression was decreased gradually during the 40 days-ES induction process (Figure S9a, Figure S10). These results confirm the intestinal epithelial barrier defects with DRD2 decrease in the colon of prolactinomas mice.

**Figure S8.** Intestinal epithelial barrier defects with DRD2 decrease in the colon of prolactinomas mice

(a) Microvilli, epithelium and cell gaps structure change of colon during the 40 days-estradiol induction process in mice by TEM. TJ: tight junction. MV: microvilli

(b) Tight junction width of intestinal barrier in colon of mice during the 40 days-estradiol induction process. n=3.

(c) Microvilli, epithelium and cell gaps structure change of colon in DRD2 (+/-) or DRD2 (-/-) mice by TEM.
(d) Tight junction width of intestinal barrier in colon of DRD2 (+/-) or DRD2 (-/-) mice. n=3.

(e-i) ZO-1, Occludin, Claudin-1, and DRD2 expressions of colon during the 40 days estradiol induction process in mice by Western blot. n=3.

(j-n) ZO-1, Occludin, Claudin-1, and DRD2 expressions of colon in DRD2 (+/-) or DRD2 (-/-) mice by Western blot. n=3. Data were expressed as mean ± S.D. The one-way ANOVA or Student’s t-test were used to perform statistical comparison by SPSS software. ^*^*p* < 0.05, ^**^*p* < 0.01, ^***^*p* < 0.001, and ^****^*p* < 0.0001.

**Figure S9.** Colon DRD2 and cell gap junction expression in prolactinomas mice

(a) The location and expression of colon DRD2 in mice induced by ES induction at different time points by immunofluorescence assay.

(b) The location and expression of colon cell gap junction protein Occludin and ZO-1 in mice induced by ES induction at different time points by immunofluorescence assay.

**Figure S10.** Colon DRD2 expression and *Escherichia coli.* in mice

Colon DRD2 expression and *Escherichia coli.* in WT or prolactinomas mice induced by ES for 40 days or DRD2 deficiency by immunofluorescence assay.

WT: wild type mice, ES: estradiol-induced mice, DRD2(-/-): DRD2 knockout mice

**Gut-brain barrier permeability test by tracer assays in DRD2(-/-) mice**

In order to evaluate the gut-brain barrier permeability, serum-to-pituitary tracer assays by FITC-dextran in DRD2(-/-) mice has been evaluated. The results showed that fluorescent FITC-dextran was detected in the pituitary tissues of DRD2 (-/-) mice, suggesting that the blood-brain barrier permeability was increased significantly in DRD2 (-/-) mice(Figure S11a,b). Considering that intraperitoneal injection bypasses physiological gut barrier penetration, two ways of administration methods intragastric gavage or intrarectal instillation of fluorescent *Escherichia coli* were performed in DRD2(-/-) mice respectively. The results showed that fluorescent *Escherichia coli* were both detected in the pituitary tissues of DRD2(-/-) mice whenever treated by intragastric gavage or intrarectal instillation of *Escherichia coli* (Figure S11c,d)*.* Therefore, our study claims that gut-derived *E. coli* translocate to the pituitary.

**Figure S11. Gut-brain barrier permeability test by tracer assays in DRD2(-/-) mice.**

(a) Serum-to-pituitary tracer assays by fluorescent FITC-dextran assay in DRD2 (-/-) mice. Arrow indicate FITC-dextran.

(b) Content detection of FITC-dextran 4000 in the pituitary tissues of DRD2 (-/-) mice. n=5.

(c) *Escherichia coli* assay of the pituitary tissues in DRD2 (-/-) mice after intragastric gavage of fluorescent *Escherichia coli .* n=3. Arrow indicate fluorescent *Escherichia coli.*

(d) *Escherichia coli* assay of the pituitary tissues in DRD2 (-/-) mice after intrarectal instillation of fluorescent *Escherichia coli.* n=3. Arrow indicate fluorescent *Escherichia coli.*

Data were expressed as mean ± S.D. The one-way ANOVA or Student’s t-test were used to perform statistical comparison by SPSS software. ^*^*p* < 0.05, ^**^*p* < 0.01, ^***^*p* < 0.001, and ^****^*p* < 0.0001.

**GSDMD is highly expressed in** **brain endothelial cell (bECs) of pituitary gland**

Gsdmd was highly enriched in bECs of brain vasculature cells and microglia cells in mice by single-cell RNA sequencing.^[33,34]^ Rencent reports demonstrate GSDMD may integrate various inflammatory stimuli in bECs, resulting in BBB leakage and brain inflammation.^[35]^ To study whether the blood pituitary barrier defects was regulated by GSDMD, colocalization analyses of pituitary GSDMD and the endothelial marker CD31 were performed in mice by immunofluores- cence assay. The results revealed that GSDMD is highly expressed in bECs of pituitary adenoma, and pituitary GSDMD expression of mice induced by ES or with DRD2 deficiency were both increased significantly than that of control mice (Fig. S12). Similar results were also confirmed in tumor tissues from nonfunctional pituitary adenoma and prolactinomas patients (Fig. S13). These data suggest GSDMD pore form in bEC of pituitary gland and thus leakage through the BBB in prolactinomas mice.

**Figure S12.** GSDMD is highly expressed in brain endothelial cell (bECs) of pituitary gland in mice.

Left: Images of GSDMD expression in the CD31+ vasculatures (green) of mice pituitary gland. Middle: high-magnification visions of the fluorescent signals in the boxed area of the left panel. Right: Distribution of anti-GSDMD/anti-CD31 fluorescent intensity (grayscale: 0–250) along the white lines marked in the middle images. All data are representative of three independent experiments.

**Fig. S13.** GSDMD is highly expressed in brain endothelial cell (bECs) of human pituitary gland.

Left: Images of GSDMD expression in the CD31+ vasculatures (green) of human pituitary tumor (nonfunctional or prolactinomas). Middle: high-magnification visions of the fluorescent signals in the boxed area of the left panel. Right: Distribution of anti-GSDMD/anti-CD31 fluorescent intensity (grayscale: 0-250) along the white lines marked in the middle images. All data are representative of three independent experiments.

***Escherichia coli* were phagocytosed by** **microglial cell expressing DRD2 of pituitary gland**

The bacterial colonization of *Escherichia coli* in the pituitary adenoma was determined with 16S RNA FISH assay. The results showed that *Escherichia coli* were phagocytosed by microglial cell of pituitary tumor from both prolactinomas patients or mice induced by ES or DRD2 deficiency. DA transmit neural signals in brain neurons, but also can regulate the immune functions of cells.^[36]^ As resident macrophages in the central nervous system, microglial cells play a critical role in its development, homeostasis, and response to injury and infection in the brain.^[37,38]^ More, there are both DA D1 and D2 classes of receptors expressed in the microglial cells. DA regulates the synthesis of microglial nitric oxide, and promotes the migration of these cells by acting on DA receptors in microglial cells.^[39,40]^ To confirm DRD2 expression in pituitary gland, DRD2 and IBA-1 protein of tumor tissues from prolactinomas patients, ES-induced mice or DRD2(-/-) mice were determined by immunofluorescence assay. The results suggested that bacteria *Escherichia coli* were phagocytosed by the microglial cell expressing DRD2 of pituitary glands (Fig. S14a, b). More, the role of other immune cells (e.g., macrophages, T cells) have been investigated. The results showed that pituitary macrophage marker CD68 or T cell marker CD3 were both not expressed and activated in tumor tissues from prolactinomas patients by immunofluorescence assay (Figure S15). Therefore, *E. coli* activates only microglial cells not macrophages or T cells and the MAPK pathway to induce tumorigenesis.

**Figure S14.** *Escherichia coli* were phagocytosed by microglial cell expressing DRD2 of pituitary gland.

(a) Pituitary *Escherichia coli*, DRD2 and IBA-1 expression of tumor tissues from prolactinomas patients by 16S RNA FISH and immunofluorescence assay.

(b) Pituitary *Escherichia coli*, DRD2 and IBA-1 expression of tumor tissues from control, ES-induced or DRD2(-/-) mice by 16S RNA FISH and immunofluorescence assay.

Left: Images of *Escherichia coli* or DRD2 expression in the IBA-1+ microglia (yellow). Right: high-magnification views of the fluorescent signals in the boxed area in the left panel.

**Figure S15.** *Escherichia coli* were not phagocytosed by other immune cells of pituitary gland.

Pituitary *Escherichia coli*, CD68 and CD3 expression of tumor tissues from nonfunctional pituitary adenoma or prolactinomas patients by 16S RNA FISH and immunofluorescence assay.

Left: Images of *Escherichia coli* in the CD68+ macrophages (green) or CD3+ T cells (yellow). Right: high-magnification views of the fluorescent signals in the boxed area in the left panel.

**Microglial depletion experiments in DRD2(-/-) mice**

The microglial depletion experiments were performed in DRD2(-/-) mice by intraperiton- eally injection with microglia remover (PLX5622) for 10 days. The results revealed that the pituitary microglia cells were cleared mostly, and the pituitary PRL and MAPK (p-P38/P38, p-JNK/JNK, p-ERK/ERK) expressions were decreased significantly in pituitary tissues of DRD2 (-/-) mice by PLX5622 compared with DRD2(-/-) mice (Figure S16). Therefore, our study confirmed that *E. coli* drive the tumorigenesis of pituitary adenoma by activating microglia GSDMD/HMGB1/MAPK pathway.

**Figure S16.** Microglial depletion experiments in DRD2(-/-) mice.

(a) Pituitary microglia cells assay in DRD2(-/-) mice after intraperitoneally injection with microglia remover (PLX5622) by IBA-1 immunofluorescence assay. n=3. Red indicate microglia cells*.*

(b-l) Pituitary PRL and MAPK (p-P38/P38, p-JNK/JNK, p-ERK/ERK) expression in DRD2(-/-) mice after intraperitoneally injection with microglia remover (PLX5622). n=3. Data were expressed as mean ± S.D. The one-way ANOVA or Student’s t-test were used to perform statistical comparison by SPSS software. ^*^*p* < 0.05, ^**^*p* < 0.01, ^***^*p* < 0.001, and ^****^*p* < 0.0001.

**Prolactinomas with barrier damage** **in** **elderly mice**

The blood-brain barrier (BBB) limits entry of blood-derived products, pathogens, and cells into the brain, which is very important for normal brain operation and neuronal functioning. Previous study have showed an age-dependent BBB breakdown in the hippocampus, a region involved in learning and memory that is defective early in Alzheimer's disease.^[41]^ The BBB breakdown was found in the hippocampus of human brain during normal aging.^[42]^  The age of most patients with pituitary adenoma are relatively old in the clinical, whether this disease is associated with age-dependent BBB breakdown is unknown. In this study, the effect of age-dependent BBB breakdow and intestinal epithelial barrier defects on prolactinomas was studied by using one month old and twelve month old mice. The serum PRL(*p*<0.0001) level of twelve-month old mice was much higher than that of one-month old mice, which was same with serum LPS (*p*<0.05), LBP (*p*<0.05), IL-18 (*p*<0.05), and GSDMD(*p*<0.01) levels (Figure S17a-f). Then, the dissociated pituitary tissues of mice were used to culture bacteria in Mueller-Hinton agar or blood agar plate respectively, showing that there were massive of bacteria in the pituitary cells of twelve-month old mice (Figure S17g). Furthermore, bacteria residing in the cytosol of pituitary prolactin-producing tumor cells in twelve-month old mice was also found by TEM assay (Figure S17i). The MS identification results confirmed the bacteria were *Escherichia coli*(Figure S17h)*.*

Furthermore, pituitary DRD2 expression was decreased significantly (*p*<0.05) as same as cell gap junction protein ZO-1, Occludin, and Claudin-1 (all *p*<0.05), while NLRP3, GSDMD, PRL,

HMGB1, p-JNK/JNK, p-ERK/ERK, and p-P38/P38 expression were increased remarkably (Figure S17j, all *p*<0.05). The BPB of pituitary gland became diffusely tightly connected, with damaged endothelial cells and no tissue structure, and the colonic microvilli became sparse, with damaged epithelium and widened cell gaps in twelve month old mice by TEM analysis (Figure S17k,m). And colon DRD2, ZO-1, Occludin, and Claudin-1expressions of twelve month old mice were decreased significantly than those of one month old mice (Figure S17l, all *p*<0.05). More, the damage of blood brain barrier were destroyed, and *Escherichia coli.* from gut entered into the pituitary tissue in twelve month old mice **(**Figure S18 a-c). Surprisingly, there were also *Escherichia coli.* in the cerebral cortex, hypothalamus, cerebellum and midbrain tissues of twelve-month old mice **(**Figure S18 d-e). These results suggest that the intestinal barrier and blood pituitary barrier are both damaged while DRD2 decrease in twelve month old mice, and *Escherichia* from the gut translocate into pituitary gland promoting the formation of prolactinomas.

**Figure S17.** Prolactinomas with barrier damage in elderly mice.

(a-f) Serum PRL, LPS, LBP, IL-1β, IL-18 and GSDMD levels in one month old and twelve month old mice. n=6.

(g) Bacterial culture of dissociated pituitary tissues by Mueller-Hinton agar or blood agar plate. n=3.

(h) Microbial species identification of cultured pituitary tissue sample from one month old and twelve month old mice by MS.

(i) BPB structure change of pituitary gland in one month old and twelve month old mice by electron microscopy. TJ: tight junction.

(j) Pituitary DRD2, cell gap junction and MAPK protein expression in one month old and twelve month old mice by Western blot. n=3.

(k) BPB defect change of pituitary gland in one month old and twelve month old mice by observing evans blue color. Left: brain tissue, right: pituitary gland

(l) Colon DRD2 and cell gap junction protein expression in one month old and twelve month old mice by Western blot. n=3.

(m) Microvilli, epithelium and cell gaps structure change of colon in one month old and twelve month old mice by TEM. TJ: tight junction. MV: microvilli. Data were expressed as mean ± S.D. The one-way ANOVA or Student’s t-test were used to perform statistical comparison by SPSS software. ^*^*p* < 0.05, ^**^*p* < 0.01, ^***^*p* < 0.001, and ^****^*p* < 0.0001.

**Fig. S18.** Damage of blood pituitary barrier in elderly mice.

(a) BPB defect change of pituitary gland in one month old and twelve month old mice by observing evans blue color. Left: brain tissue, right: pituitary gland. n=3.

(b) *In vitro* tracing of *Escherichia coli.* labeled with fluorescent in one month old and twelve month old mice. n=3.

(c) Pituitary *Escherichia coli.* determination by immunofluorescence assay in one month old and twelve month old mice. n=3.

(d) Bacteria culture of cerebral cortex, hypothalamus, cerebellum and midbrain tissues from one month old and twelve month old mice. n=3.

(e) *Escherichia coli* assay of cerebral cortex, hypothalamus, cerebellum and midbrain tissues from one month old and twelve month old mice by 16S RNA FISH. n=3.

**Figure S19.** DRD2 and tight junction expression of human tumor samples from prolactinomas and non-functional pituitary adenoma patients by Western blot. Data were expressed as mean ± S.D. The one-way ANOVA or Student’s t-test were used to perform statistical comparison by SPSS software. ^*^*p* < 0.05, ^**^*p* < 0.01, ^***^*p* < 0.001, and ^****^*p* < 0.0001.
